# Brucellosis: Bacteriology, pathogenesis, epidemiology and role of the metallophores in virulence: a review

**DOI:** 10.3389/fcimb.2025.1621230

**Published:** 2025-07-08

**Authors:** Ghassan Ghssein, Zeinab Ezzeddine, Sima Tokajian, Charbel Al Khoury, Hussein Kobeissy, Jose-Noel Ibrahim, Christelle F. Iskandar, Hussein F. Hassan

**Affiliations:** 1 Department of Laboratory Sciences, Faculty of Public Health, Islamic University of Lebanon, Beirut, Lebanon; 2 Department of Biological Sciences, School of Arts and Sciences, Lebanese American University, Beirut, Lebanon; 3 Department of Nutrition and Food Sciences, Faculty of Agricultural and Food Sciences, American University of Beirut, Beirut, Lebanon; 4 Department of Nutrition and Food Science, School of Arts and Sciences, Lebanese American University, Beirut, Lebanon

**Keywords:** brucellosis, zoonotic disease, Brucella, livestock, public health threats

## Abstract

Brucellosis is a recognized zoonotic disease caused by various *Brucella* species with significant economic and animal welfare ramifications worldwide. The spread of brucellosis from domestic livestock and wild animals, as well as its emergence in new regions, present novel epidemiological challenges. The consumption of unpasteurized milk and dairy products from unsanitary farms in endemic areas poses a serious risk to public health from brucellosis. Determining the accurate prevalence of brucellosis, particularly in regions with persistently high prevalence, basically requires careful and frequent surveillance. Furthermore, transmission and detection of the illness in non-endemic areas have become more complex due to global human and animal migration as well as the trade in animal products. This review presents an updated understanding of brucellosis, covering its classification and taxonomy, pathogenesis, diagnosis and treatment approaches, epidemiology, available control and prevention measures, antimicrobial resistance and the role of metal uptake in bacterial virulence. It highlights the consequences of brucellosis for global health and underscores the need for continuous research, knowledge sharing, and interdisciplinary cooperation for effective disease control and prevention.

## 1 Introduction

Brucellosis, frequently referred to as Malta fever, is a significant zoonotic infection that affects humans as well as domestic and wild animals. The infection can impact ungulates, marine mammals, rodents, carnivores, and primates. Bacteria of the genus *Brucella* were identified and named after David Bruce (1855–1931) as the primary cause of infertility and reproductive losses, with a tendency to induce mastitis, placentitis, and neonatal pneumonia (Gorvel, 2008). The classic or core *Brucella* species are *Brucella* abortus, *Brucella* canis, *Brucella* melitensis, *Brucella* neotomae, *Brucella* ovis, and *Brucella* suis. These are traditionally recognized as the main species within the genus *Brucella*. They are known for their host preferences and are often associated with specific animal populations (Occhialini et al., 2022). *Brucella* species are also recognized as zoonotic, meaning they could infect humans and cause serious illness (Adams, 2002). Recently, a group of taxonomists merged the brucellae with the primarily free-living, phylogenetically related *Ochrobactrum* spp. in the genus *Brucella* but Genetic comparison of Brucella spp. and Ochrobactrum spp. erroneously included into the genus Brucella confirms separate genera (De Figueiredo et al., 2015; Moreno et al., 2023). Despite significant advancements in scientific research and practical knowledge, in addition to almost a century of intensive research in many countries, brucellosis remains a persistent or reemerging zoonosis, causing significant economic losses, morbidity in humans, and prolonged poverty globally. Currently, the genus has ten species after new isolates from aquatic mammals (*B. pinnipedialis* and *B. ceti*), humans (*B. inopinata*), and common voles (*B. microti*) were identified. Although pseudogenes that affect host adaptability might play a role, the mechanisms underlying host preference are still unknown. The burden of Brucellosis on animals worldwide is significant. According to conservative estimates, more than 300 million of the 1.4 billion cattle globally are afflicted with the pathogen. Animals with brucellosis exhibit many clinical symptoms, including abortion (De Figueiredo et al., 2015). Various species of *Brucella* can infect humans unintentionally. Human illness typically arises via eating contaminated animal products, such as raw milk and cheese, or from direct contact with the tissues or blood of infected animals. Human brucellosis usually manifests as a high, wavy fever. However, chronic brucellosis can cause encephalomyelitis, endocarditis, hepatitis, arthritis, and orchitis, among other illnesses affecting numerous host organs (Dean et al., 2012; Young et al., 2014). The most frequent complication of Brucellosis is arthritis. The illness’s variety of symptoms makes diagnosis more difficult. Even in the majority of high-income countries, brucellosis has eluded systematic eradication efforts for over a century, and there remains no licensed human vaccine available (Bernués et al., 1997). *Brucella* species are classified as category B pathogens and possible bioterrorism agents due to their ability to aerosolize and the low infectious dose required for transmission. The estimated financial impact of a *Brucella* bioterrorism attack is second only to anthrax and tularemia, with an infectious dose of 10 to 100 organisms. Furthermore, the possibility of intentional release presents a direct threat to public health in metropolitan areas, one that cannot be addressed by the standard method of immunizing animals (Cutler et al., 2005). This review highlights all the aspects related to *Brucella* spp, including bacteriology, pathophysiology, diagnostics, treatment, control and prevention, virulence factors, and metal acquisition.

## 2 Classification and taxonomy

By comparing distinct sets of features, taxonomy, and nomenclature are examples of artificial systems designed to improve comprehension of the relationships between species. Due to the prior use of nonsystematic methodologies, such as common names, which led to unclear and erroneous nomenclature, early attempts at bacterial taxonomy were problematic. To address these issues, a decision was taken to move away from the taxonomic classification of higher organisms and begin a new framework for bacterial nomenclature. This was accomplished by creating new regulations intended to streamline classification and prevent pointless or confusing modifications.

The genus *Brucella* is a member of the class *Alphaproteobacteria* of the phylum *Proteobacteria*, belonging to the family *Brucella*ceae (family III), together with *Mycoplana* and *Ochrobactrum*, of the order *Rhizobiales* (Ruan, 2013). Families of organisms that are either plant or mammalian pathogens or symbionts are included in the class *Alphaproteobacteria*. The genera *Ehrlichia, Rickettsia*, and *Bartonella* are among the *Alphaproteobacteria* species that infect mammals; they are all transmitted through vectors. The small genome sizes of these organisms are compatible with their obligate intracellular survival, although this characteristic does not often characterize insect vector-based transmission, such as that of Coxiella. *Brucella* differs from most genera in the order *Rhizobiales* by having a streamlined genome in comparison to plant pathogens and the ability to infect mammalian cells—a characteristic that it shares only with *Bartonella*. However, there are significant differences between *Brucella*, a facultative intracellular pathogen, and *Bartonella*, an obligate intracellular pathogen. Firstly, compared to *Bartonella* spp., the genome of *Brucella* spp. is 50–100% larger and retains more metabolic processes commonly found in plant pathogens. The capacity to use plant-based compounds metabolically is consistent with the ability to persist in the soil for up to 10 weeks (Paulsen et al., 2002). The three genera share an environmental niche, as evidenced by the recent discovery of *Brucella microti* in soil (Scholz et al., 2008). The comparatively large genome size of *Brucella* species reflects their ability to exist in a variety of conditions, which may involve host adaptation. Variations in optimal growth conditions and cell surface structures (such as the cell wall) of different host species may also be reflected in the specific mechanisms for the uptake and intracellular growth of mammalian pathogens. Therefore, both *Bartonella* and *Brucella* may have acquired the ability to infect mammalian hosts, at least in part. As a result, it is possible that their nucleotide composition (i.e., G+C content) differs from that of genes conserved from progenitor organisms. Genes encoding secretion systems, adhesins, invasins, and polysaccharide biosynthesis are among the several candidates for this function (DelVecchio et al., 2002; Halling et al., 2005).

Nonetheless, it is likely that genes involved in the absorption or penetration of mammalian cells existed in ancestral species and were eliminated by plant diseases. In this scenario, the genes would not show characteristic nucleotide compositions and would need to be identified using more straightforward methods. An analysis of the genomes of several *Brucella* species reveals that during adaptation to an intracellular environment, genes lose their functionality due to the development of pseudogenes (Chain et al., 2005). More recently, it has been shown that adaptation to an intracellular lifestyle is linked to horizontal gene transfer specific to *Brucella* species, which is linked to significant virulence factors (Wattam et al., 2009). The most striking example is the suggestion that tissue tropism and host range in the nonzoonotic pathogen *Brucella ovis* were restricted by the inactivation of genes related to urease production, cell envelope construction, and nutrient uptake (Tsolis et al., 2009). These studies, however, do not distinguish between adaptation that occurs later in life and coevolution with a primary host.

The apparent adaptability of *Brucella* species to certain hosts has appropriately been the center of speculation regarding their origin. Based on currently observed host preferences, coevolution between *Brucella* species and their chosen hosts is a logical starting point. However, the minimal genetic variance found across *Brucella* species and the overall genetic variety seen between host species do not align with this straightforward conclusion. Although it is obvious that host and agent do not always evolve at the same rate, the general similarities found in *Brucella* species that have adapted to their hosts support either limited genetic flexibility or a more recent adaptation. Foster and colleagues concluded that most *Brucella* species diverged from a common ancestor (similar to *B. ovis*) within the past 86,000–296,000 years (Foster et al., 2009). This time frame unquestionably predates the domestication of livestock hosts, but it is by no means close to the time of host species divergence (Blair Hedges and Kumar, 2003). Their molecular clock, based on single nucleotide polymorphisms in 13 different *Brucella* genomes representing the original six species, supports this estimate. In conclusion, adaptation to and final preference for primary hosts do not appear to have played a significant role in the divergence of the *Brucella* spp.

It is important to note that the host preference of *Brucella* species is not as strict as it might seem. *In vitro* or in the wild, animals other than their primary host are susceptible to infection by *Brucella* bacteria. These infections, though, seem to be self-limiting. Furthermore, serious *Brucella* infections, including abortion storms, are exclusively caused by infection with the preferred species in regions where cattle and goats or cattle and swine overlap. The most well-studied example to date involves cattle that encountered feral pigs that were found to have contracted *Brucella* suis infection. Although bacteria were shed in the milk of infected animals, the infection was not transmissible, and infected cows gave birth to normal, healthy calves (Ewalt et al., 1997). Thus, the concept of host-specific adaptation is still a relevant area for further investigation.

Due to the seeming inability to reconcile genetic diversity with the wide range of phenotypes used to distinguish species and subspecies within the genus, there has been considerable interest in the taxonomy and nomenclature of *Brucella* (Whatmore, 2009). *Brucella* species have been identified since the late 19th and early 20th centuries, mostly from the host species from which they were isolated and in which they induce persistent and severe infection. David Bruce identified *Brucella melitensis* as the causative agent of illness in British soldiers stationed in Malta in 1887 (Ficht, 2010). But Themistocles Zammit deserves recognition for proving that goat milk was the cause of human illness (Wyatt, 2005). *Brucella* species were also discovered to be connected to other hosts in the following decades, such as *Brucella abortus* in cattle, *Brucella sui*s in pigs, *Brucella canis* in dogs*, Brucella ovis* in sheep, and *Brucella neotomae* in the desert wood rat (Kurmanov et al., 2022). There are discernible variations in the severity of sickness caused by these agents when compared in a single host, such as humans, despite the fact that they have all been summarily categorized as class III biohazards. While *B. ovis, B. neotomae, and B. canis* are not classified as select agents, *B. abortus, B. melitensis, and B. suis* are regarded as significant public health hazards (Ficht, 2010).

## 3 Bacteriology

*Brucella* is a small Gram-negative, intracellular coccobacilli bacterium that lacks spores, flagella, and capsules (Al Dahouk et al., 2013). David Bruce discovered and isolated *Brucella* for the first time in 1886 from the spleens of soldiers who died from “Maltese fever” (Mantur et al., 2007). The cell wall of *Brucella* is made up of two membranes. *Brucella’s* outer membrane is composed of a phospholipid layer, outer membrane proteins, and lipopolysaccharide (LPS). The LPS of *Brucella* is made up of three components. First, the toxic component of the molecule is lipid A, a hydrophobic lipid moiety that is anchored in the membrane. Second, the non-repeated phosphorylated polysaccharide, known as the core, contributes to the outer membrane’s non-permeability. The core is divided into two distinct regions: the outer core, a branched pentasaccharide composed of glucose, galactose, and *N-acetyl*-_D_-glucosamine; and the inner core, which is characterized by sugars like _L_*-glycero-_D_-manno*-heptose and the essential eight-carbon sugar acid 3-deoxy-_D_-*manno*-octulosonic acid (Kdo). The third oligosaccharide is the O antigen, a repetitive oligosaccharide that varies significantly even among strains of the same species. It serves as a significant virulence factor for *Brucella* as well as an essential antigen that stimulates the body’s immunological response (Cardoso et al., 2006). *Brucella* primarily infects macrophages and trophoblast cells. It parasitizes host cells through a particular molecular mechanism and influences host cell death, which in turn facilitates host cell autophagy, establishing an environment that is conducive to its survival and propagation in the host cells (Weynants et al., 1996).

**Figure 1:**
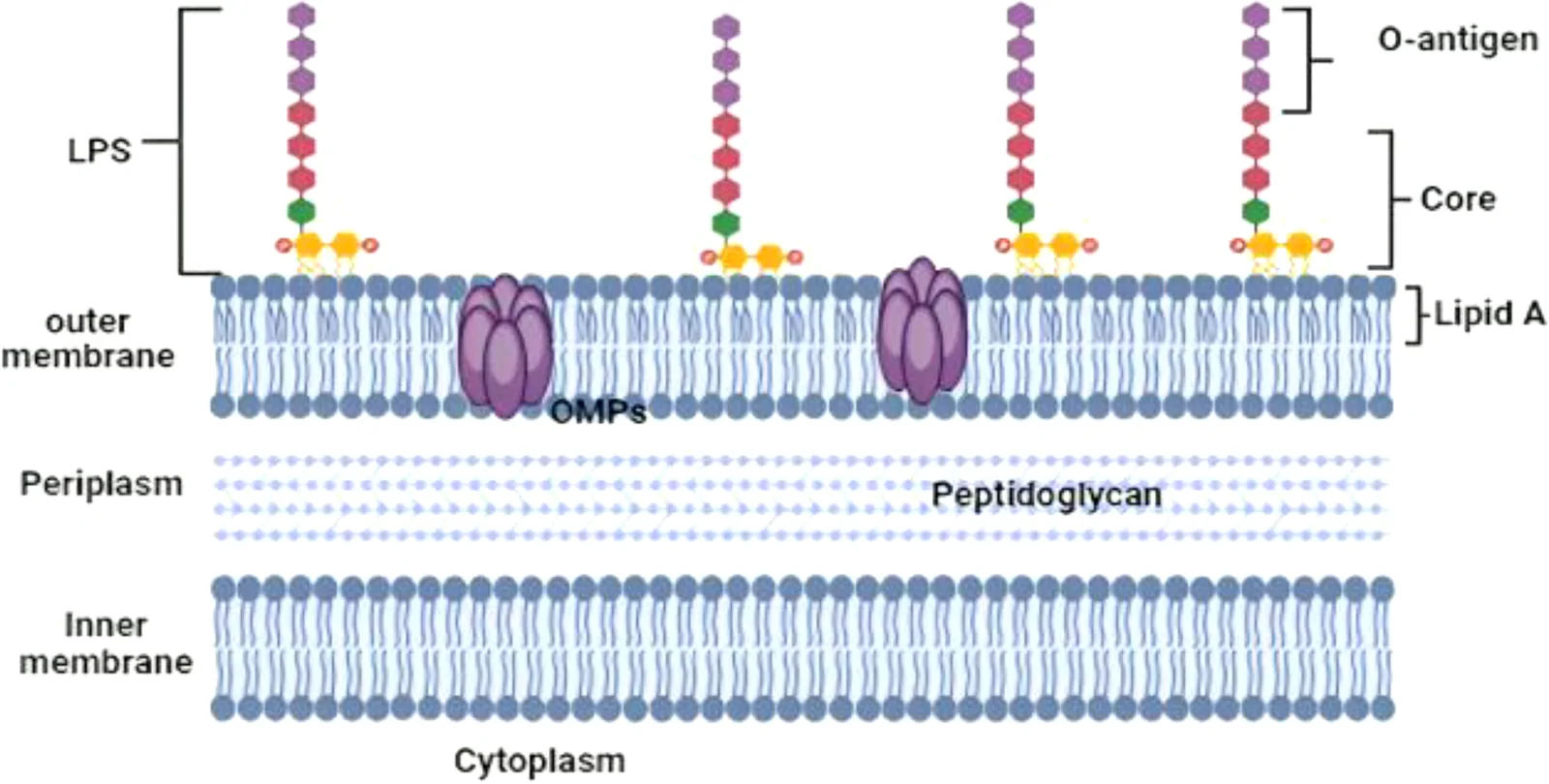
Schematic illustration of the *Brucella* cell wall.

There are various structural variations between the enterobacteria frequently seen in alphaproteobacteria and the structure of *Brucella* lipid A. In addition to glucosamine, the presence of diaminoglucose implies that there are two populations of core lipid A molecules. Long saturated molecules (C16:0 to C18:0) and the exceptionally long-chain molecule 27-hydroxy-octacosanoate (27-OH-C28:0) are found in the fatty acid chains. The lack of phosphate, neutral carbohydrates, and ethanolamine is another feature. Additionally, the core region’s structure differs from that of the enterobacterial core. Mannose, glucose, quinovosamine (2-amino-2,6-dideoxy-_D_-glucose), non-substituted Kdo, and trace amounts of other sugars are the main constituents of the core region. The absence of the heptose region is another notable anomaly. All species of *Brucella*, except *B. canis* and *B. ovis*, have smooth LPS. The O-chain structure of *Brucella* species is a linear homopolymer of 4,6-dideoxy-4-formamido-α-_D_-mannose. Either 1,2 or 1,3 glycosidic links can be used to join individual units, and the proportion of these linkages in the O-polysaccharide varies between species of *Brucella*. Three primary epitopes—the A, M, and common epitopes—identified by monoclonal antibodies result from the specific arrangement of these connections and are present in all *Brucella* sp*ecies*. Numerous remarkable characteristics of the *Brucella* LPS envelope, including its permeability to hydrophobic substances and resistance to EDTA and cationic peptides such ad polymyxin, are caused by the bacterium’s unique chemical structure. Due to these structural variations, *Brucella* lipid A is significantly less toxic than enterobacterial lipid A, which is another notable effect. There are seven *Brucella* outer-membrane proteins (OMPs) that are exposed on the surface. These consist of the Ompl0, Ompl6, and Ompl9 lipoproteins, as well as the Omp25, Omp2b, and Omp31 proteins (Liu, 2015). The schematic illustration of the *Brucella* cell wall is shown in **Figure 1**.

### 3.1 Biology of *Brucella*

*In vivo*, *Brucella* rapidly propagate over the mucosal epithelial layer (Rossetti et al., 2013) and are taken up by dendritic cells (DCs) and mucosal macrophages. Through cellular tropism, *Brucella* is able to live and multiply inside competent phagocytic cells, avoid detection by the host immune system, and spread to target organs, such as the reproductive tract, fetal lungs, reticuloendothelial system, and placental trophoblasts in pregnant women (Adams, 2002). To comprehend the adhesion, internalization, intracellular trafficking, survival, and replication of *Brucella* in vulnerable hosts, *in vitro* investigations were employed as models. In order to internalize itself, *Brucella* initiate a zipper-like process on the surface of mucosal epithelial cells (Rossetti et al., 2012). *Brucella* binds to sialic acid and sulfated residue-containing binding molecules on the surface of epithelial cells, which are activated before and/or upon contact. However, these binding molecules are still not fully understood.

Binding stimulates small GTPase activity, which initiates a signaling cascade that reorganizes the actin cytoskeleton and induces a rearrangement of the host cell membrane along the pathogen’s surface, thereby enhancing invasion. Shortly after contact, entry occurs, necessitating the complete activation of a mitogen-activated protein kinase signaling cascade. *Brucella* can live and multiply among inactivated phagocytic cells for up to 72 hours *in vitro*. *In vivo*, they can traverse the epithelium by undermining the function of the mucosal epithelial barrier, which enables *Brucella* to undergo transepithelial migration. This connection simultaneously triggers a minor innate immune response and modest proinflammatory activity (Barquero-Calvo et al., 2007). After being transferred across the epithelium, *Brucella* are taken up by mucosal phagocytic cells, where less than 10% of the phagocytized bacteria make it through a period of adaptation.

*Brucella* reduce, modify, or hide their pathogen-associated molecular patterns to evade immune recognition and trigger an immune response. On the other hand, some Toll-like receptors (TLRs; primarily TLR2, TLR4, and TLR9) start a limited intracellular signaling that activates the transcription factor NF-κB to control the expression of inflammatory cytokine genes (Oliveira et al., 2008) albeit at a 10-fold lower level than that seen with enterobacteria. *Brucella* live in a unique vacuole called the *Brucella*-containing vacuole (BCV) inside mononuclear phagocytic cells. They alter intracellular trafficking in this vacuole and convert it into a replicative compartment called a brucellosome. Research suggests that the BCV’s microenvironment has a restricted supply of nutrients12, to which *Brucella* quickly adjusts upon invasion (Köhler et al., 2002).

In order to adapt to low oxygen tension, the pathogen first increases amino acid catabolism, switches to alternative energy sources, and quantitatively reduces gene expression and protein synthesis involved in anabolic metabolism (Lamontagne et al., 2009). The development of a type IV secretion system (T4SS) early after infection is crucial for intracellular survival and multiplication inside mammalian cells in an *in vitro* brucellosis infection model. However, *in vivo* research has shown that while the T4SS is required for prolonged persistence, it is not necessary for invasion, systemic dispersion, or the development of the initial infection (Roux et al., 2007).

Invading *Brucella* that survive the adaptation phase during infection progressively restore the expression of important genes encoded by metabolic processes. This transcription-translation reactivation primarily affects cell membranes, transporters, and iron metabolism (Lamontagne et al., 2009). *Brucella* reproduce in tandem with the restoration of essential processes, such as the expression of virulence genes, which are occasionally strictly regulated by quorum-sensing molecules (Rambow-Larsen et al., 2008; Weeks et al., 2010). When an infection occurs, infected mononuclear phagocytic cells undergo significant transcriptional alterations during the adaptation stage. After 12 hours, when *Brucella* replication starts, the modifications return to normal. After adapting to the intramacrophage environment, *Brucella* prolongs its intracellular persistence indefinitely. This leads to the infection of desired targeted cells or tissues, including the reticuloendothelial system, endothelium, male genitalia, fetal lungs, skeletal tissues, and placental trophoblasts, as well as systemic metastasis. In order to give a more comprehensive systems biology description of the pathogenesis of brucellosis at the level of the entire host organism, there is currently a dearth of evidence describing the interaction of *Brucella* with these target cells and tissues (Carvalho Neta et al., 2008; Delpino et al., 2009).

## 4 Virulence factors and pathogenesis

### 4.1 Type IV secretion system

The type IV secretion system (T4SS) of *Brucella* has been the most extensively investigated factor influencing its virulence (DelVecchio et al., 2002). The 11 proteins that comprise the *Brucella* T4SS are a lytic transglycosylase (VirB1) that remodels the bacterial cell peptidoglycan layer during T4SS assembly, two ATPases (VirB4 and VirB11) that supply energy to drive effector secretion and eight proteins that comprise the core of the transporter (VirB2, VirB3, and VirB5 through VirB10) (Watarai et al., 2002c; Höppner et al., 2004; Carle et al., 2006). The operon that contains the T4SS genes is conserved in all strains of *Brucella*, and the virB mutants of *B. abortus, B. melitensis, B. suis, B. canis, B. ovis, B. microti*, and *B. neotomae* are significantly reduced in both natural and cultured hosts (Sieira et al., 2000; Patey et al., 2006; Sivanesan et al., 2010; Kang et al., 2019). Genes located outside of the virB operon also encode *Brucella* proteins that aid in the assembly and functionality of the T4SS. One such protein, VirJ, is a periplasmic protein whose exact role is still unknown, but it is necessary for the correct assembly of the T4SS and interacts directly with T4SS substrates that have a periplasmic intermediate during their export (Del Giudice et al., 2016). One of the main functions of the T4SS is to regulate the intracellular trafficking of the *Brucella*-containing vacuoles in host macrophages, preventing the bacteria from being killed and degraded in phagolysosomes (Comerci et al., 2001; Watarai et al., 2002a; Celli et al., 2003).

**Figure 2:**
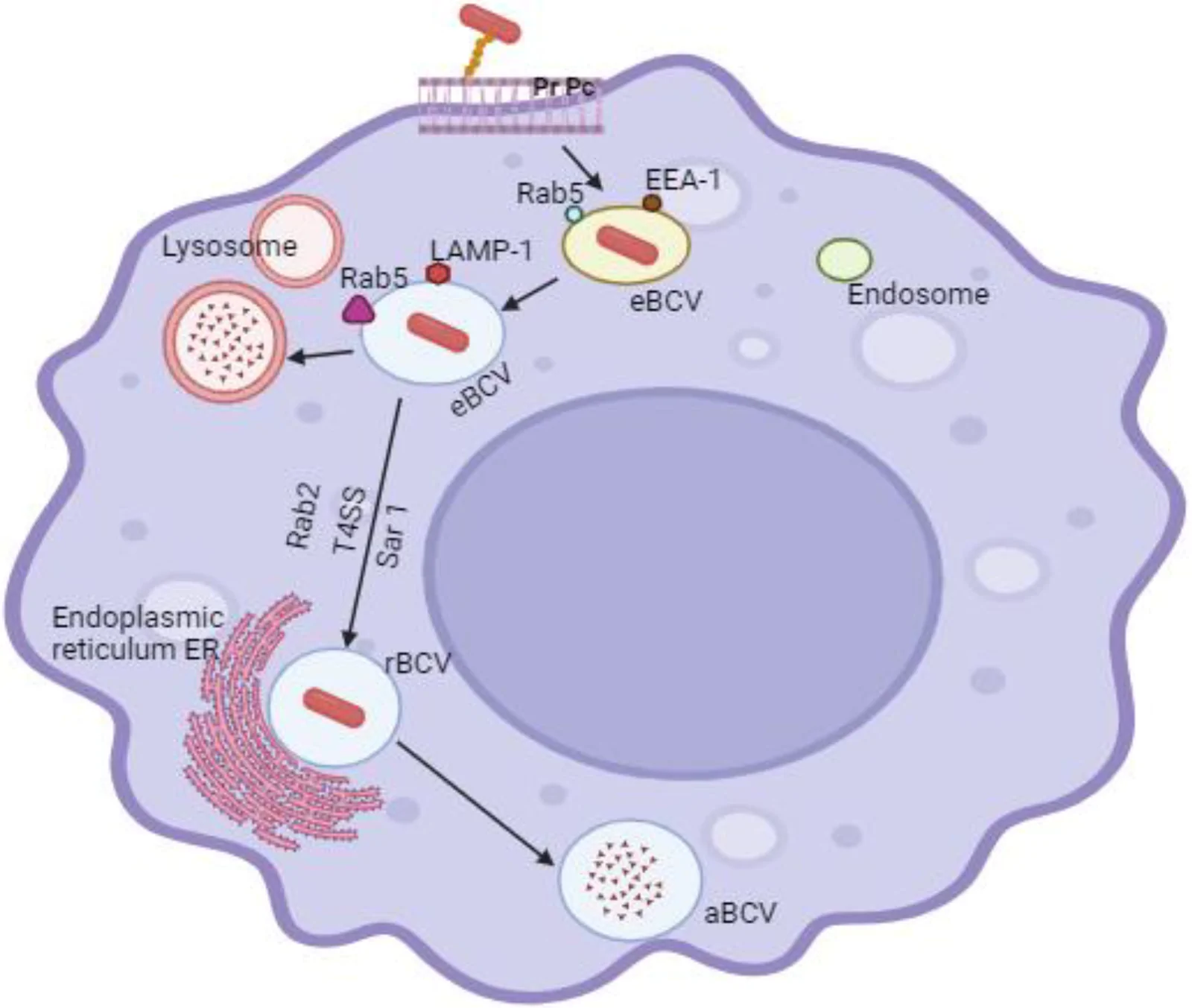
Through lipid rafts, *Brucella* interacts with macrophage cell membranes to penetrate host cells and produce *Brucella*-containing vacuoles (BCV), which are encircled by phagocytic vesicles. Eight to twelve hours after *Brucella* enters the cell, the virus (BCV) develops the endosomes in the membrane-bound vacuoles, creates acidified endosomes, and receives certain host marker molecules by contact with lysosomes (Lys) and endosomes. Currently, endosomal *Brucella* containing vacuole (eBCV) is the term used to refer to BCV. The Type IV secretory system (T4SS) receives membranes derived from the Golgi apparatus and the endoplasmic reticulum (ER) by mediating the connection between the effector protein and the ER exit site as BCV grows and matures. The eBCV acquired Lys marker molecules (such Rab7, LAMP-1, etc.) after losing the early host marker molecules.

Initially, *Brucella* was identified as a facultative internal bacterial parasite that could multiply in phagocytes, such as granulocytes, macrophages, and dendritic cells (DC), as well as epithelial, fibroblastic, and trophoblastic cells (Copin et al., 2012). Through lipid rafts, *Brucella* interacts with macrophage cell membranes and enters the host cell to produce *Brucella*-containing vacuoles (BCV), which are encircled by phagocytic vesicles (Köhler et al., 2003). Eight to twelve hours after entry, BCV matures into endosomes within the membrane-bound vacuoles, acidifies, and acquires host marker molecules through contact with lysosomes (Lys) and endosomes. Currently, the term “endosomal *Brucella*-containing vacuole” (eBCV) is used to refer to a BCV. The Type IV secretory system (T4SS) receives membranes derived from the Golgi apparatus and the endoplasmic reticulum (ER) by mediating the connection between the effector protein and the ER exit site as BCV grows and matures. The eBCV acquired Lys marker molecules (such as Rab7, LAMP-1, etc.) after losing the early host marker molecules (Von Bargen et al., 2012). When the BCV avoids Lys degradation, it will eventually reach the ER and fuse there in a way that depends on Rab2 and Sar1 (Celli et al., 2003). At this stage, the BCV is referred to as a replicative *Brucella*-containing vacuole, or rBCV. rBCV will change into autophagic *Brucella*-containing vacuole (aBCV) at a later stage of infection (**Figure 2**). The aBCV will not continue to develop and destroy cells at this time. At this point, *Brucella* has finished the intracellular circulation, and the organism uses lysis and nonlysis methods to release pathogens (Starr et al., 2012).

By interacting with lipid rafts on the plasma membrane, *Brucella* can mediate its internalization into phagocytes and to facilitate interaction with host cells. Glycosphingolipids and cholesterol found in lipid rafts have the ability to stimulate biological processes associated to membranes, including membrane fusion, transmembrane signaling, and the production of polybasic membrane complexes (Cutler et al., 2005). LPS can stop complement-mediated bacterial lysis and host cell apoptosis, and it is a crucial molecule in the interaction between *Brucella* and lipid rafts in the cell (Jiménez De Bagüés et al., 2004). *Brucella* invades cells through lipid rafts, and it has been demonstrated that the class A scavenger receptor (SR-A) and the prion protein (PrPc) are involved in this process (Watarai et al., 2003; Kim et al., 2004).

Specific lipid rafts contain prion proteins and SR-A, which are receptors for heat shock protein 60 (HSP60) and LPS. The early survival of *Brucella* in macrophages can be efficiently reduced by lipid raft destruction, suggesting that lipid raft introduction is a prerequisite for the early survival of bacteria (Naroeni and Porte, 2002). *Brucella* enters the cell to form a phagosome and take part in the endocytosis pathway, but it can be easily detached from the phagosome, suggesting that the lipid raft-mediated signaling pathway plays a role in *Brucella*’s early survival (Porte et al., 2003). aBCV is required by *Brucella* to finish its intracellular life cycle and cell-to-cell dissemination during intracellular circulation (Starr et al., 2008). The conversion of rBCV to aBCV starts when the ER Beclin1 and PI3K form a complex, although this process gradually slows down as ATG14L is consumed. The effector protein BspB interacts with the conserved oligomeric Golgi (COG) to regulate COG-dependent transport, reorient Golgi-generated vesicles to BCV, boost rBCV production, and enhance *Brucella* intracellular proliferation (Jiao et al., 2021).

Modulating the host immune response is another mechanism that the *Brucella* T4SS increases virulence (Rolán and Tsolis, 2007; Roux et al., 2007; Rolán and Tsolis, 2008). For example, the host cell ER chaperone BiP interacts with the T4SS effector VceC (De Jong et al., 2013). In *Brucella*-infected cells, this interaction results in ER stress and an unfolded protein response (UPR), which in turn promotes the production of the inflammatory cytokines interleukin 6 (IL-6) and tumor necrosis factor alpha (TNF-α). The synthesis of these cytokines by macrophages in response to VceC leads to the formation of granulomas, which promotes the persistence of infection. Researchers hypothesize that this T4SS effector may be crucial to transmission in natural hosts because VceC-mediated inflammatory cytokine release by placental trophoblasts also causes host cell death and fetal disease in the pregnant mice model (Keestra-Gounder et al., 2016; Byndloss et al., 2019).

The T4SS effectors BtpA and BtpB, in contrast to VceC, block dendritic cells’ ability to produce inflammatory cytokines by disrupting the TLR-Myd88-MAL signaling pathway (Salcedo et al., 2008; Sengupta et al., 2010; Alaidarous et al., 2014). It has been suggested that the T4SS effectors’ dual ability to stimulate and inhibit host immune responses enables *Brucella* to induce a response in the host that is beneficial to their long-term intracellular persistence. This balance ensures enough immunopathology to aid in their spread to other hosts, but not strong enough to cause sterilizing immunity and end the infection (De Jong et al., 2010).

### 4.2 Lipopolysaccharide

*Brucella* strains, like most Gram-negative bacteria, produce lipopolysaccharide (LPS), which is essential for maintaining the integrity of their cell envelope (Roop et al., 2021). They have a smooth LPS (S-LPS) composed of a polysaccharide O-chain, core, and lipid A, with two major exceptions: B. ovis and B. canis, which naturally produce a crude LPS lacking the O-chain. There is ample evidence to support the significance of the O-chain for the pathogenicity of naturally occurring smooth *Brucella* strains (Monreal et al., 2003; Roop et al., 2021). By shielding smooth *Brucella* strains from the complement’s bactericidal effects (González et al., 2008; Ouahrani-Bettache et al., 2019) and the antimicrobial peptides they encounter when interacting with host phagocytes, the LPS O-chain is one way that it contributes to virulence or by acting as an adhesin. Smooth *Brucella* strains can enter mammalian cells *via* an endocytic pathway that circumvents the broad fusion of BCVs with lysosomes, thanks to the interaction of the O-chain with lipid rafts on the surface of these cells (Porte et al., 2003). Before the involvement of the T4SS effectors, this entry point is the first necessary step in the replication of BCV by smooth strains (Naroeni and Porte, 2002; Watarai et al., 2002b).

Because this pathway of entry promotes modest levels of proinflammatory cytokine production in macrophages and dendritic cells, O-chain-mediated uptake of smooth *Brucella* strains also plays a significant role in immune evasion (Jiménez De Bagüés et al., 2004; Billard et al., 2007). In addition to being resistant to being broken down by macrophages, smooth LPS secreted by *Brucella* strains into BCVs also forms complexes with major histocompatibility complex class II (MHC-II), which prevents these phagocytes from presenting antigens to T lymphocytes (Forestier et al., 2000; Lapaque et al., 2006a). Inhibiting caspase 2-mediated cell death in these phagocytes is yet another mechanism whereby the O-chain-mediated entrance of smooth *Brucella* strains into macrophages adds to virulence (Gross et al., 2000; Fernandez-Prada et al., 2003; He et al., 2006). Although the exact processes underlying this suppression are unknown, smooth strains’ ability to prolong macrophage life expectancy probably improves the macrophages’ resistance to immunological clearance and ability to spread to many organs within their mammalian hosts (Pei et al., 2006; Chen and He, 2009).

Since lipid A is the part of LPS that the host pattern recognition receptor Toll-like receptor 4 (TLR4) recognizes, and since the lipid A of many Gram-negative bacteria generates potent inflammatory reactions, lipid A’s are sometimes referred to as “endotoxins” (Bryant et al., 2010). The production of low endotoxin activity lipopolysaccharides (LPS) by *Brucella* strains has long been reported (Duenas, 2004; Tumurkhuu et al., 2006). The fact that *Brucella* lipid A contains very-long-chain fatty acids (VLCFAs), in contrast to its enteric counterparts, may be one reason for its reduced inflammatory response in certain strains of the bacteria (Velasco et al., 2000; Ferguson et al., 2004). These VLCFAs most likely stop the *Brucella* lipid A from interacting with TLR4 in the same potent ways as other bacterial lipid A molecules (Lapaque et al., 2006b). Remarkably, human neutrophils are similarly susceptible to early cell death caused by the *Brucella* lipid A. Macrophages and dendritic cells then consume dead neutrophils that harbor intracellular *BrucellaBrucella* through non-inflammatory mechanism. This has been suggested as one further tactic that *Brucella* strains may use to evade the host immune system’s recognition in the early phases of infection (Barquero-Calvo et al., 2015).

**Figure 3:**
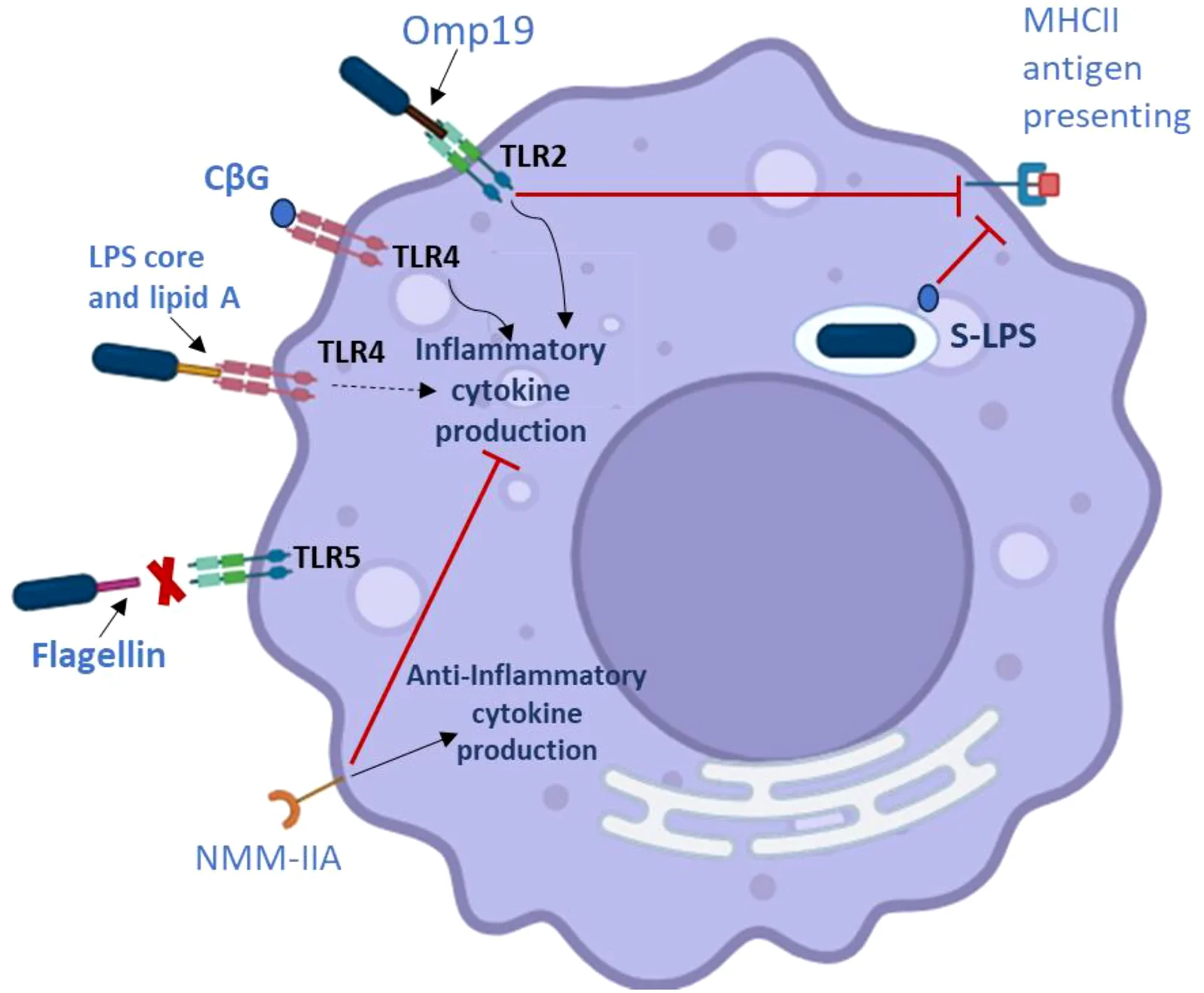
Factors related to *Brucella* virulence that affect macrophages’ ability to adjust the host immunological response. ∣, inhibition; →, activation. The dashed arrow shows that while the *Brucella* LPS produces a reduced inflammatory response, it does not signal through the TLR4 pathway robustly. The red X means that TLR5 does not identify the *Brucella* flagellin.

The primary structural function of the LPS core in many bacteria is to connect lipid A with the O-chain. It serves the same purpose in *Brucella*, but more recently, research has demonstrated that the LPS core is crucial in helping these bacteria evade recognition by the host immune system (Fontana et al., 2016; Salvador-Bescós et al., 2018). To be more precise, the *Brucella* generates a core structure with a side chain of lateral oligosaccharides that sterically covers lipid A and prevents it from binding to TLR4 on host dendritic cells and macrophages (**Figure 3**). The resistance of both smooth and rough *Brucella* strains to complement and bactericidal peptide killing appears to be influenced by this lateral side chain and the positive charge it provides on the LPS core (Soler-Lloréns et al., 2014).

*Brucella* species that infect domestic animals, along with many strains that infect wildlife, share a highly conserved organization and composition of LPS biosynthesis genes. The absence of the O-chain from the LPS of *B. ovis* and *B. canis* is caused by well-documented genomic deletions (Zygmunt et al., 2009). However, some of the so-called early diverging *Brucella* strains that were isolated from human diseases and found in amphibians use the operon *rmlABCD*, which consists of four genes, to produce an LPS with an O-chain based on rhamnose rather than the perosamine O-side chain found in all other smooth *Brucella* strains (Wattam et al., 2012). It has been hypothesized that the acquisition of the genes encoding this latter kind of LPS O-chain was crucial to the evolution of *Brucella* strains as mammalian pathogens, given the proven significance of the perosamine O-chain in virulence (Wattam et al., 2009). It is interesting to note that serologic research indicates that the LPS cores of some early divergent strains might differ from those of the 200 typical *Brucella* strains.

### 4.3 Omp25/Omp31

**Figure 4:**
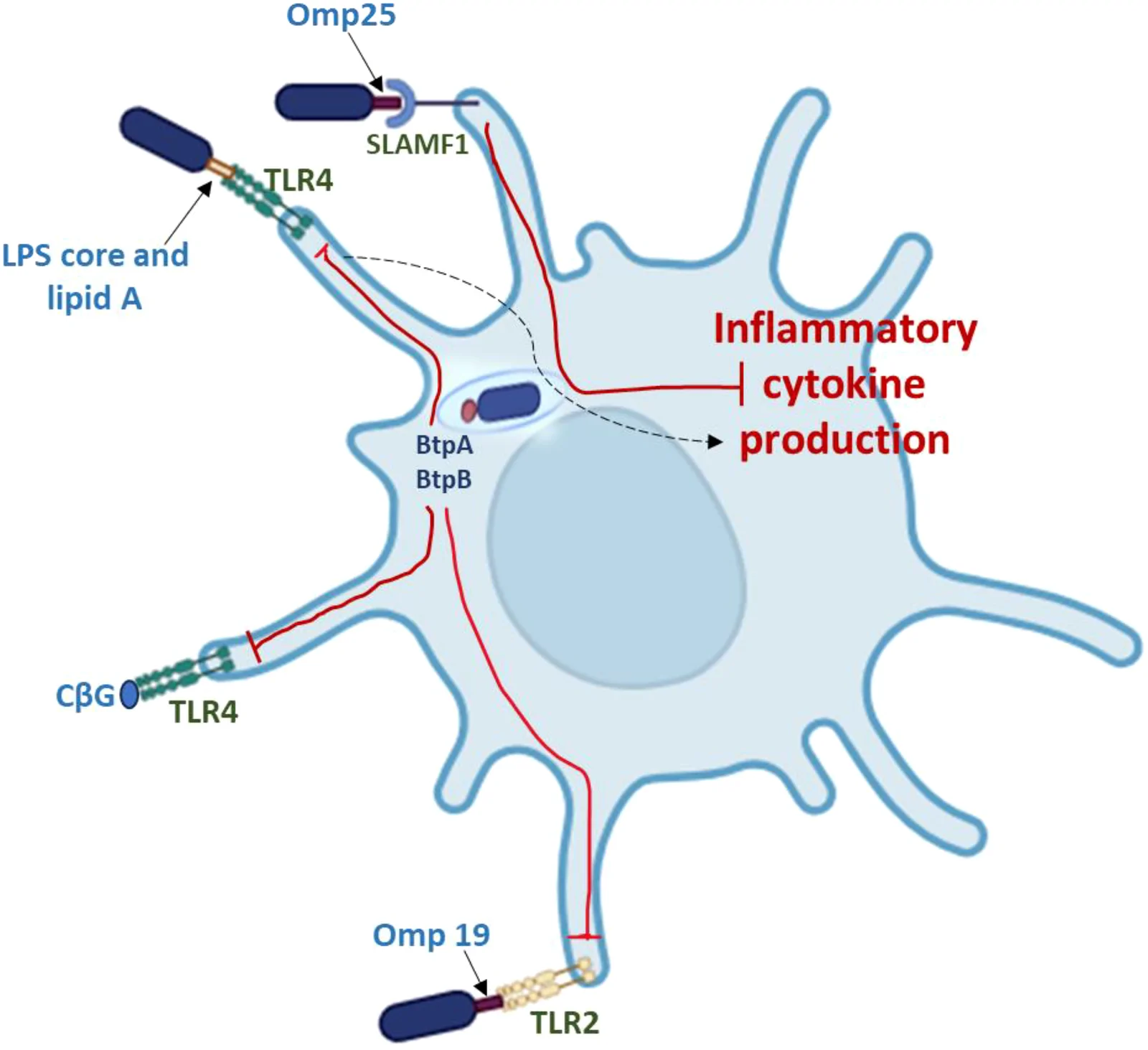
Factors associated with *Brucella* virulence that affect dendritic cells’ capacity to control the host immunological response. ∣, inhibition. The dashed arrow shows that while the *Brucella* LPS produces a reduced inflammatory response, it does not signal through the TLR4 pathway robustly.

Omp25, Omp25b, Omp25c, Omp25d, Omp31, Omb31p, and Omp22 are a family of highly conserved outer membrane proteins (OMPs) produced by *Brucella* strains that are crucial for preserving the integrity of their cell envelope (Cloeckaert et al., 2002). These β-barrel OMPs protect the bacteria against complement and other antimicrobial peptides found in the host when they function in tandem with the LPS O-chain. Their virulence contributions seem to be particularly significant for naturally occurring rough strains such as *B. ovis* (Edmonds et al., 2002; Martín-Martín et al., 2008). It has been demonstrated that *B. abortus* and *B. melitensis* omp25 mutants are attenuated in mice (Edmonds et al., 2002) and natural hosts (Edmonds et al., 2001), and that a *B. melitensis*omp31 mutant is attenuated in both mice and cultured mammalian cells (Verdiguel-Fernández et al., 2017; Fernández et al., 2020). The *Brucella* Omp25/Omp31 proteins have been shown to facilitate direct contacts between the *Brucella* and mammalian cells that are crucial for virulence, in addition to their structural roles in preserving cell envelope integrity. Some of the divergent phenotypes observed in virulence experiments for *Brucella* omp25 and omp31 mutants may be explained by these latter roles. In smooth strains (Manterola et al., 2007), where the LPS O-chain appears to be the predominant determinant in mammalian cell entry, there is no evidence that Omp25d and Omp22 perform this function. In contrast, Omp25d and Omp22 play significant roles in *B. ovis* entry into mammalian cells (Martín-Martín et al., 2008). The smooth strain *B. abortus’*s Omp25 protein also directly interacts with dendritic cells’ SLAMF1 protein on their surface, preventing them from maturing and releasing inflammatory cytokines (Degos et al., 2020) (**Figure 4**). Jubier-Maurin et al. (2001a) initially documented Omp25’s ability to suppress TNF-α production during *Brucella* infection; however, the molecular mechanisms of this function have only recently been determined. It is unclear exactly how these other hypothesized Omp25/Omp31 functions contribute to virulence, but there is evidence that the *Brucella* Omp25 and Omp31 proteins have the ability to modify other elements of host cell function during infection (Jubier-Maurin et al., 2001a; Zhang et al., 2016; Cui et al., 2017; Luo et al., 2018). The great degree of conservation of the Omp25/Omp31 proteins throughout the *Rhizobiaceae* family is one of its intriguing characteristics. The Omp25/Omp31 orthologs are also crucial for the productive relationships of other alphaproteobacteria with their respective eukaryotic hosts. For example, *Agrobacterium tumefaciens*’ wild-type pathogenicity in plants requires the Omp25 ortholog AopB (Jia et al., 2002). *Bartonella henselae* hbp knockdown strains are attenuated in endothelial cell cultures (Liu et al., 2012), and the *Bartonella* heme-binding proteins (Hbps) are likewise Omp25/Omp31 orthologs (Minnick et al., 2003). Remarkably, it has also been demonstrated that the *Brucella* Omp31b binds hemin *in vitro*, and the *B. suis* gene that codes for this protein is iron-controlled (Jubier-Maurin et al., 2001a).

It has not been demonstrated that any one *Brucella* species can synthesize all seven Omp25/Omp31 proteins (Martín-Martín et al., 2009). Large genomic deletions have resulted in the loss of genes encoding distinct Omp25/Omp31 proteins in some species, such as *B. abortus* and *B. ovis* (Pei et al., 2006). Other *Brucella* species have different patterns of Omp25/Omp31 production, which seem to be caused by smaller genetic abnormalities (Martín-Martín et al., 2009). The tight coordination of the expression of the omp25 and omp31 genes in response to environmental stimuli and physiological cues by the global regulators BvrRS, VjbR, and CtrA (Viadas et al., 2010) is consistent with the important function that this family of OMPs plays in the fundamental physiology and pathogenicity of *Brucella* strains.

### 4.4 Omp10, Omp16, and Omp19

Three outer membrane lipoproteins (Omp10, Omp16, and Omp19) are produced by different strains of *Brucella* (Tibor et al., 1999). The peptidoglycan-associated lipoprotein (Pal), which is largely conserved in Gram-negative bacteria, has a homolog in Omp16 (Godlewska et al., 2009). These proteins interact with the constituents of the Tol complex and are essential for maintaining the structural integrity and functionality of the outer membrane. Omp16’s role as a pal homolog is consistent with the fact that it is an important gene in *Brucella* (Sidhu-Muñoz et al., 2016; Sternon et al., 2018; Zhi et al., 2020). Conversely, Omp19 is the most well-studied lipoprotein produced by *Brucella*. Through its interactions with TLR2, purified Omp19 has strong immunomodulatory activities that affect a wide range of host cells. These interactions have been linked to *Brucella*’s ability to evade host immune responses as well as their ability to induce immunopathology in specific tissues, including bone and the central nervous system (Coria et al., 2016; Velásquez et al., 2017) (**Figures 3**, **4**). Furthermore, phenotypic analysis of an omp19 mutant *B. abortus* reveals that Omp19 shields the parental strain against lysosomal proteases encountered during intracellular residence in host macrophages and those encountered in the intestinal tract following oral infection (Pasquevich et al., 2019). Omp19 also shares significant amino acid homology with bacterial protease inhibitors (Ibañez et al., 2015), and it prevents proteases from degrading Omp25, another immunomodulatory protein. Omp10 homologs are only found in *Brucella* and a small number of other alphaproteobacteria (Cloeckaert et al., 1999), in contrast to Omp16 and Omp19, which exhibit homology to proteins from other bacteria. The biological role of this Omp is uncertain. Interestingly, the equivalent *B. ovis* mutants do not exhibit significant attenuation in mice (De Souza Filho et al., 2015), although *B. abortus* omp19 and omp10 mutants do (Sidhu-Muñoz et al., 2016). However, the inability to create double mutants of *B. ovis* omp10 omp19 raises the possibility that these proteins share a physiological role (Sidhu-Muñoz et al., 2016).

In addition to the previously mentioned proteins, *Brucella* species contains more proteins including Omp2a/2b, and BP26. The omp2a and omp2b gene products are homologous outer membrane proteins that function as porins, meaning they form channels in the outer membrane that allow small molecules to pass through. These proteins are crucial for the bacterial outer membrane, playing a role in nutrient and toxin transport. Variation in the omp2 locus, particularly in the omp2b gene, is linked to *Brucella* species identification and evolutionary adaptations (Paquet et al., 2001). *Brucella* BP26, also known as Omp28, is a 26 kDa periplasmic protein of *Brucella* species, a major immunodominant antigen that is widely used in diagnostic and vaccination efforts against brucellosis. BP26 is a target for antibodies in infected animals and humans, and its presence is a key indicator in the diagnosis of the disease (Kim D. et al., 2013).

### 4.5 Autotransporter adhesins

Autotransporter (AT) adhesins are of significant importance in facilitating the attachment of numerous bacterial pathogens to mammalian cells (Henderson et al., 2004). *Brucella* is known to possess five distinct AT adhesins. Among these, OmaA and BmaC are categorized as type I monomeric ATs (Posadas et al., 2012), whereas BtaE and BtaF fall under type II trimeric ATs (Muñoz González et al., 2019). Furthermore, BigA (264) showcases shared structural domains with the *Escherichia coli* adhesin intimin, representing an inverse AT adhesin (265). BmaC specifically binds to fibronectin present on the surface of host cells (260), while BtaE and BtaF have an affinity for hyaluronic acid (Ruiz-Ranwez et al., 2013). However, the specific receptors for OmaA and BigA remain unidentified. Interestingly, *Brucella* mutants lacking these AT adhesins demonstrate decreased attachment to epithelial cells, while still displaying wild-type intracellular replication in cultured macrophages. Moreover, these mutants exhibit attenuated virulence in mice when administered via intragastric or nasal routes, as opposed to intraperitoneal delivery. These experimental findings suggest that the primary role of AT adhesins is to enhance the attachment of *Brucella* to the host at mucosal surfaces during the initial phases of infection. Notably, BigA exhibits a preference for eukaryotic cell junctions (Leo et al., 2015; Czibener et al., 2016), a mechanism also employed by various bacterial pathogens to traverse mucosal barriers within the host (Ruch and Engel, 2017). Additionally, a double mutant of *B. suis* lacking btaE and btaF displays significantly higher attenuation in mice compared to single mutants of btaE or btaF, highlighting the complementary roles these adhesins play in virulence (Ruiz-Ranwez et al., 2013). Besides their attachment function, there is evidence suggesting that BtaF aids in protecting *B. suis* from the bactericidal effects of serum (Ruiz-Ranwez et al., 2013).

BmaC, BtaE, and BtaF are predominantly localized at a specific pole of the bacterial cell (Posadas et al., 2012). The concentration of these AT adhesins at the new pole, in conjunction with the observation that *Brucella* cells in the G1 phase of the cell cycle are the predominant infectious form (Deghelt et al., 2014), has led researchers to propose that these adhesins collectively form an adhesive pole on the *Brucella* cell (Van Der Henst et al., 2013; De Bolle et al., 2015). Only a fraction of *Brucella* cells in planktonic cultures express these adhesins, indicating that the corresponding genes may only be optimally expressed upon exposure to a host-specific stimulus, such as interaction with mammalian cells. This observation aligns with the regulatory control of several AT-encoding genes by the quorum-sensing regulator VjbR (Uzureau et al., 2010) and the global regulator MucR (Pirone et al., 2018), as well as the intricate regulatory network known to govern btaE expression in *B. abortus* (Kleinman et al., 2017). The variability in the genes encoding AT-type adhesins within *Brucella* (Sieira et al., 2017) suggests functional overlap among these adhesins, as previously reported (Ruiz-Ranwez et al., 2013). Therefore, a comprehensive exploration of the roles of *Brucella* AT adhesins across different species and strains, utilizing mutants with multiple disrupted genes, is crucial for a precise evaluation of their contributions to virulence.

### 4.6 Cyclic β-1,2- _D_-Glucan

**Figure 5:**
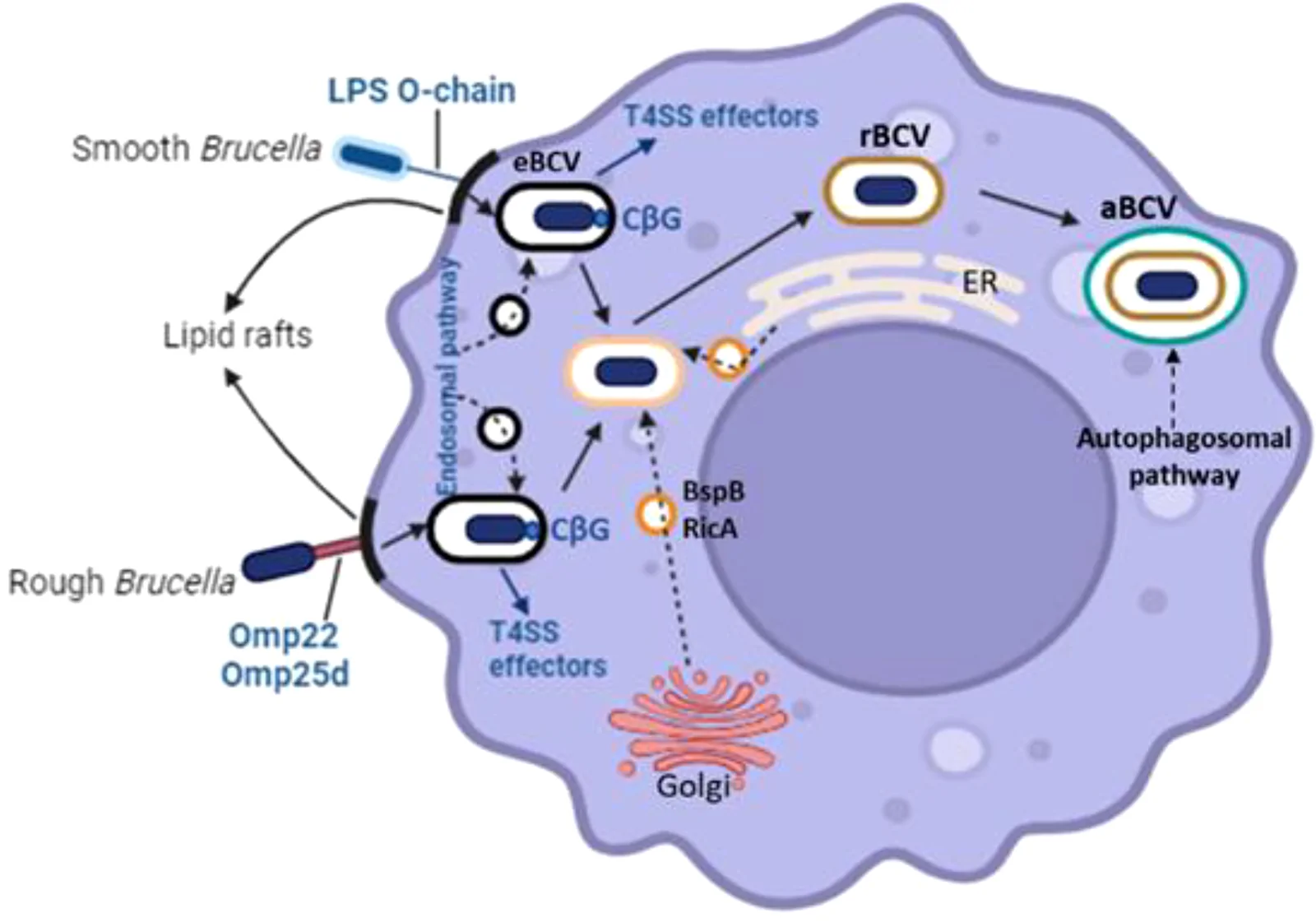
The contributions of various components such as the T4SS effectors, the LPS O-chain, Omp22, Omp25d, and cyclic β-1,2-Dglucan (CbG) are pivotal in the formation of the replicative *Brucella*-containing vacuole within host macrophages. The membrane vesicles, depicted as empty black and orange circles, are involved in trafficking from the endolysosomal pathway, endoplasmic reticulum, and Golgi apparatus towards the *Brucella*-containing vacuoles (BCVs).

Numerous Gram-negative bacteria synthesize polysaccharide polymers and release them into their periplasmic space, where they execute various physiological functions (Bontemps-Gallo and Lacroix, 2015). *Brucella* spp. and other alphaproteobacteria discharge a cyclic polymer comprised of 17 to 20 glucose units, referred to as cyclic β-1,2-glucan (CβG), into their periplasmic compartment (Iñón De Iannino et al., 1998). Within Sinorhizobium and Agrobacterium, the synthesis of CβG is controlled by osmotic conditions, indicating a potential function of this compound in osmotic protection (Breedveld and Miller, 1994). However, in *Brucella*, the production of CβG is not influenced by osmotic factors (De Iannino et al., 2000), and experimental data indicate that this polysaccharide has a minor impact as an osmoprotectant in these bacteria (Roset et al., 2014). Nonetheless, CβG plays a crucial function in the virulence of *Brucella* (Briones et al., 2001). Research utilizing *B. abortus* CβG synthase mutants and purified CβG has shown that this compound disrupts lipid rafts on the vacuoles containing *Brucella*, thereby impeding their interactions with lysosomes (Arellano-Reynoso et al., 2005) (**Figure 5**). Furthermore, CβG has been demonstrated to significantly affect the ability of macrophages and dendritic cells to generate both proinflammatory and anti-inflammatory cytokines (Martirosyan et al., 2012; Roset et al., 2014; Degos et al., 2015; Guidolin et al., 2018) (**Figures 3**, **4**). The mechanism by which CβG is released from its periplasmic site in *Brucella* cells for these biological functions *in vivo* remains unclear. Nevertheless, CβG appears to possess dual roles in virulence. It is pivotal in the intracellular transportation of *Brucella* to their replicative environment within host macrophages, and it modulates the host immune response to facilitate their prolonged intracellular survival. Considering the suggested polar nature of the interaction of *Brucella* cells with their mammalian hosts (Van Der Henst et al., 2013; De Bolle et al., 2015), it is noteworthy that the CβG synthase (Cgs) and transporter (Cgt) exhibit polar distribution on the bacterial cell (Guidolin et al., 2015). The potential contribution of this polar distribution of the CβG biosynthesis and transport apparatus to virulence remains to be elucidated.

### 4.7 Flagella

In 1998, Halling reported the identification of flagellar biosynthetic genes in *B. abortus*, even though the majority of *Brucella* strains are nonmotile (Halling, 1998). Subsequent research revealed that the majority of *Brucella* strains, if not all, have the genetic potential to create flagella (Fretin et al., 2005). However, only a small percentage of strains in the BO2 clade appear to be able to employ the flagella for motility, and they lack chemotaxis genes. Nevertheless, the discovery of *B. melitensisfliF* and *flgF* mutants in signature-tagged mutagenesis screens for attenuation in pregnant goats (Zygmunt et al., 2006) and mice (Lestrate et al., 2003) raised the possibility that these genes are involved in virulence. Studies have confirmed that flagellar biosynthesis genes are necessary for the wild-type virulence of *B. melitensis* and *B. abortus* strains in the mouse model. The Letesson group demonstrated that *B. melitensis*16M does, in fact, produce a single sheathed polar flagellum that is covered by an extension of the outer membrane (Al Dahouk et al., 2017). It is interesting to note that *B. ovis* mutants devoid of genes involved in flagellar biosynthesis exhibit full virulence in mice, indicating that the role played by flagella in pathogenesis may vary depending on the strain and, perhaps, the host (Sidhu-Muñoz et al., 2020).

Modulating the host immune response is one way that the flagella seem to contribute to pathogenicity (Terwagne et al., 2013). The *Brucella* flagellin is not detected by TLR5, in contrast to flagella from many other Gram-negative pathogens. This adds to the alleged stealthiness of infections caused by *Brucella*. However, data from experiments suggest that *Brucella* flagellin enters the cytoplasm of infected host cells and triggers an inflammatory response mediated by an inflammasome, which is crucial for “limiting” the extent of *Brucella* reproduction. Therefore, it has been suggested that the flagellum is an additional virulence factor that enables *Brucella* to modify the host immune response in a manner that facilitates the formation and persistence of chronic infections. It is also possible that these appendages, like those of other closely related alphaproteobacteria, have unidentified functions in pathogenesis, such as adhesins or surface attachment sensors (Hug et al., 2017). Furthermore, studies have revealed that sheathed flagella are involved in the release of outer membrane vesicles (OMVs) (Aschtgen et al., 2016), which is an intriguing relationship given the roles that OMVs have been suggested to play in host-pathogen interactions during *Brucella* infections (Pollak et al., 2012). Furthermore, sheathed flagella are relatively uncommon in bacteria.

While production of the polar flagellum in *B. melitensis*16M has only been observed *in vitro*, in bacterial cells grown to an early exponential phase in a rich medium, the genes involved in flagellar biosynthesis are tightly regulated in *Brucella*. These genes are readily expressed when this strain is replicating in mammalian cells (Fretin et al., 2005). A portion of the genes involved in flagellar biosynthesis in *Brucella* are expressed in phases; however, the regulatory networks governing the systematic temporal expression of these genes differ from those in other bacteria (Ferooz et al., 2011). *Brucella* flagellar gene expression is additionally regulated by the quorum-sensing regulators VjbR and BabR (also referred to as BlxR), the light-sensing regulator LovhK, the general stress response regulator RpoE1, and cyclic di-GMP-mediated signaling (Delrue et al., 2005; Kim HS. et al., 2013). Nearly all bacteria that use flagella for movement have chemotaxis genes, which enable them to regulate the direction of their flagellar rotation in response to environmental stimuli that vary in intensity (Wuichet and Zhulin, 2010). This enables them to swim away from harmful substances and toward nutrients. Given that the majority of *Brucella* strains do not appear to utilize their flagella for movement, the absence of chemotaxis genes in these bacteria is not particularly surprising. However, it does bring up some intriguing issues regarding how the motile BO2 strains navigate their native habitats.

### 4.8 Phosphatidylcholine

Prokaryotes typically lack the phospholipid phosphatidylcholine (PC), despite being a significant component of eukaryotic cell membranes (Wuichet and Zhulin, 2010). The discovery that some *Brucella* strains had PC in their cell envelopes occurred about 50 years ago (Thiele and Schwinn, 1973), which raised the possibility that the virulence of the outer membrane (OM) could be influenced by the presence of this “eukaryotic” phospholipid. *Brucella* strains use two distinct metabolic processes to produce PC: the Pcs system, which converts choline directly into PC, and the Pmt pathway, which methylates the phospholipid phosphatidylethanolamine to form PC (Herrmann et al., 2013). Independent research conducted in two distinct labs has verified that PC is crucial to *Brucella* pathogenicity. To obtain a comprehensive picture of how PC contributes to virulence, more research will be required as these investigations yielded inconsistent data about the relative contributions of the Pcs and Pmt pathways to virulence. However, the available experimental data generally indicates that the PC/PE ratio in *Brucella* strains’ outer membrane affects the bacteria’s resistance to complement and antimicrobial peptide killing, and that PC may also be involved in regulating the host’s response to infection (Bukata et al., 2008). It is also noteworthy that some other members of this group, like *Agrobacterium* and *Rhizobia*, interact with their respective plant hosts in a significant way due to PC’s incorporation into the outer membrane, which is a characteristic of alphaproteobacteria in general (Aktas et al., 2010).

### 4.9 Exopolysaccharides

Exopolysaccharides (EPSs) are polysaccharide polymers released by bacteria that are loosely linked with bacterial cells, generating an amorphous “slime layer” (Cuthbertson et al., 2009), or securely bound to the cell surface to form a capsule. EPSs are involved in several aspects of bacterial pathogenesis. They can act as adhesins and promote bacterial adhesion to eukaryotic cells (Tomlinson and Fuqua, 2009). Additionally, they can help bacteria avoid being recognized by the host’s innate and acquired immune responses (Muñoz et al., 2018), as well as shield them from the bactericidal effects of complement, neutrophils, and macrophages (Mishra et al., 2012). Furthermore, these polymers enable the formation of biofilms by bacteria, which adds to their ability to persist in the environment and mammalian hosts (Jones and Wozniak, 2017).

Genetically, *Brucella* strains are capable of producing extracellular polymeric substances (EPS) (Caswell et al., 2013), although experimental data indicate that the associated genes are highly regulated. For example, *B. melitensis*16M does not readily produce an EPS during routine *in vitro* cultivation; however, this strain produces an apparent EPS detected by calcofluor staining and forms “biofilm-like” bacterial cell aggregates in liquid culture upon disruption of a putative quorum-sensing pathway (Uzureau et al., 2007). Furthermore, a *B. melitensisvirB* mutant (Wang et al., 2010) and a *B. abortus* strain that overexpresses the glycosyltransferase WbkA (Dabral et al., 2015) have been reported to produce EPS and exhibit cellular aggregation. EPS production is also supported by reports of *Brucella* strains (Tang et al., 2019) forming “biofilms” and a *B. melitensismucR* mutant (Mirabella et al., 2013) exhibiting improved Congo red staining.

It is currently uncertain whether EPS is crucial for *Brucella* pathogenicity as the precise genes needed for EPS synthesis are still unclear. However, EPS synthesis is necessary for both the symbiotic and pathogenic relationships between rhizobia and agrobacteria and their respective plant hosts (Marczak et al., 2017; Thompson et al., 2018). The conservation of techniques used by alphaproteobacteria to maintain successful relationships with their eukaryotic hosts (Batut et al., 2004) raises the question of whether EPS synthesis contributes significantly to *Brucella* pathogenicity.

## 5 Epidemiology

### 5.1 Animal brucellosis

The incidence rate of brucellosis in developing Asian and African nations indicates that the disease is still a significant threat to both animal and human health in these areas. The prevalence of brucellosis in both Asia and African countries is 8% while it is 12% in the Indian livestock population (Suresh et al., 2022). Between 2010 and 2019, the prevalence of brucellosis in livestock ranged from 0.2% to 43.8% in cattle, 0.0% to 20.0% in goats, and 0.0% to 13.8% in sheep in many regions of the world, including sub-Saharan Africa (Djangwani et al., 2021). In Latin America, the seroprevalence of *Brucella* in bovine was 4%, with Venezuela having the highest prevalence (16%). Among regions, the highest seroprevalence is in Central America and the Caribbean islands (8% and 3%–15%, respectively) (Bonilla-Aldana et al., 2023).

*B. canis* primarily infects dogs and wild canids, but humans can also become infected. Globally, dog seroprevalence rates range from less than 1% to 15% or higher; greater rates are typically associated with stray dogs and poorer areas, most likely as a result of these communities’ higher numbers of intact canines and uncontrolled mating. In the US, *B. suis* biovars 1, 3, and 4 are detected. Biovars 1 through 3 have swine as their reservoir host; however, infections can also affect humans, dogs, cattle, and occasionally other species. Caribou and reindeer that inhabit Arctic locations, including Alaska, are infected by *B. suis* biovar 4. The eradication of *B. suis* from US commercial swine was achieved in 2011; however, the bacteria still exist in feral swine, especially in the Southern and Western Parts of the country, and it continues to pose a threat to commercial swine operations (Sandfoss et al., 2012).

Although endemic throughout Asia, the Middle East, South America, and Africa, *B. melitensis*is is not found in the United States. Three biovars and a smooth colony phenotype characterize *B. melitensis*. The reservoir hosts are sheep and goats, but it has also been reported in a wide range of other species. Similar to *Brucella* species in other host species, *B. melitensis* in small ruminants has similar clinical symptoms and modes of transmission (Garin-Bastuji et al., 2014).

Although *B. abortus* primarily affects cattle and bison, it has also been documented to affect yaks, as well as animals such as antelope, elk, sika deer, African buffalo, horses, camels, and South American camelids that encounter infected ruminants. *B abortus* biovar one has been eliminated from US cattle and domestic bison, but it was formerly thought to be the most serious livestock disease in the US, resulting in high rates of human infection through contact with sick animals or consumption of unpasteurized dairy products.

In parts of Europe, Africa, Asia, Central and South America, and Asia, *B abortus* is still enzootic. These locations also have high populations of cows, fewer economic resources, and lower implementation of control and surveillance measures (Pinn-Woodcock et al., 2023).

### 5.2 Human brucellosis

According to recent studies, there are 1.6–2.1 million new human cases worldwide each year, which is more significant than previously thought (Laine et al., 2023). High incidence rates are reported in areas with limited resources, including the Mediterranean, Middle East, Central Asia, and some parts of Africa. Among the nations with the highest documented rates of brucellosis are Iran, Kyrgyzstan, Tajikistan, Kazakhstan, Azerbaijan, Turkmenistan, Armenia, and Uzbekistan (Pal et al., 2017; Khurana et al., 2021).

Mexico and Peru have reported numerous occurrences in Latin America (Bano and Ahmad Lone, 2015). A study on the epidemiology of brucellosis in California by Fritz et al. found that the disease is most common among older Latino men and is significantly associated with the consumption of unpasteurized Mexican-style soft cheese. The most common species found in cases was *B. melitensis*. The 492 documented instances in California between 1993 and 2017 highlight the risks posed by brucellosis to human health (Fritz et al., 2021).

The yearly incidence rate for the 28 EU countries from 2017 to 2018 was 0.09 per 100,000 population (Uzunović et al., 2020). Successful intervention efforts were highlighted by the European Food Safety Authority (EFSA), which reported a decrease in brucellosis cases from 735 in 2008 to 352 in 2011 (Bano and Ahmad Lone, 2015).

In Bosnia and Herzegovina, 263 instances were examined between 2008 and 2018, down from 102 in 2008 to only three in 2018. The evidence from other international research is consistent with the epidemiological characteristics found in this investigation. In particular, there was a noticeable male preponderance; patients were mostly from rural areas or had prior animal contact; the most affected age group was 25–49 years old (Bano and Ahmad Lone, 2015; Ali et al., 2018; Uzunović et al., 2020).

Since 1989, 80,000 instances of brucellosis have been documented annually, and the disease is found across much of Iran. Healthcare personnel in Iran have reportedly come into unintentional contact with *Brucella* strains while administering standard animal vaccinations (Alavi and Motlagh, 2012).

According to a study by Holt et al. (2021), brucellosis, a zoonotic illness caused by the *Brucella* species, is prevalent in rural India, with a seroprevalence rate of 15.1%. Due to close human-animal contact, this finding highlights the disease’s prevalence in areas with a high concentration of agriculture and livestock production, which facilitate disease transmission.

According to a study by Nawaz et al. on the epidemiology of brucellosis in Punjab, Pakistan, the seroprevalence was 13.13%, with higher rates in males aged 25 to 40 years (Sero-epidemiology, 2021).

With a national average annual incidence of 3.0 per 100,000, a four-year study found that the incidence of brucellosis varied throughout China. The rate more than doubled in Inner Mongolia, leading to a greater incidence rate in Northern China, while it significantly dropped in Xinjiang. Interestingly, men in this region aged between 45 and 64 are more than twice as likely to be impacted by women (Tao et al., 2021).

In many parts of the world, particularly sub-Saharan Africa, brucellosis is endemic. The prevalence of brucellosis in livestock varied from 0.2% to 43.8% in cattle, 0.0% to 20.0% in goats, and 0.0% to 13.8% in sheep, according to studies published between 2010 and 2019. Prevalence of human brucellosis in sub-Saharan Africa varies from 0% to 55.8%, indicating the substantial frequency of infection in this region (Djangwani et al., 2021).

A study performed in Algeria revealed that 15% of the veterinarians interviewed had contracted brucellosis during their professional practice. Direct, unprotected exposure to infected animals and/or their products, mainly during intervention for placental retention, recurrent encounters with brucellosis-infected farms, and unprotected handling of anti-Brucella vaccine appear to be the most common modes of contamination. The lack of protective equipment worn by veterinarians in their daily practice could be an important risk factor for brucellosis in these professionals. The lack of training in the handling of the antibrucellosis vaccine has made it a potential factor for brucellosis contamination, resulting in several cases of professional contamination (Lounes et al., 2022).

## 6 Antimicrobial resistance

The penetration of most antibiotics is restricted by the intracellular location of *Brucella* in reticuloendothelial cells and their preferred locations, such as bone. To treat brucellosis, antimicrobial regimens containing quinolones, doxycycline, rifampicin, streptomycin, and aminoglycosides are administered either alone or in combination (Ariza et al., 2007). There are several instances of brucellosis relapses after therapy, ranging from 5% to 15% in uncomplicated cases, and treatment failure occurs often (Del Pozo and Solera, 2015). In areas of the world where brucellosis is endemic, such as Egypt, Qatar, Iran, Malaysia, and China, antibiotic resistance in *Brucella* has recently been observed (Del Pozo and Solera, 2015).

Brucellosis in the Nile River Basin countries (Egypt, Sudan, Ethiopia, and Tanzania) is highly prevalent and endemic. There are several factors behind the failure of eradication of *Brucella* in these countries. In Ethiopia for example, brucellosis is one of the top five neglected zoonotic diseases in the country. According to several studies, the distribution and prevalence of bovine and human brucellosis in Ethiopia varies among regions in terms of animal production and management systems, community living standards and awareness levels. The disease has major zoonotic and economic implications for rural communities, particularly pastoralists (Erkyihun et al., 2022).The lack of cooperation between policymakers, health officials, veterinary sectors, and farmers is the key reason that impedes the control and prevention strategies in brucellosis endemic countries. The ‘test-and-slaughter’ strategy and the pasteurization of milk, which have been implemented successfully in the more economically developed countries, might not be the optimal control tools in most African countries due to scarcity of resources (Hikal et al., 2023).

Khan et al. studied antibiotic resistance of *Brucella* spp. isolated from animal populations in several locations in Egypt. In total, 34 *Brucella* isolates were discovered in the lymph nodes, milk, and fetal abomasal contents of sick cattle, buffaloes, sheep, and goats across nine regions. Chloramphenicol, ciprofloxacin, erythromycin, gentamicin, imipenem, rifampicin, streptomycin, and tetracycline were among the clinically used antimicrobial agents used for antimicrobial susceptibility testing. Using molecular techniques, the genes and mutations linked to antibiotic resistance in *Brucella* isolates were verified. Eight *B. abortus* biovar 1 and twenty-one *B. melitensis* biovar 3 were among the 29 *Brucella* isolates that were identified and typed. The ciprofloxacin, erythromycin, imipenem, rifampicin, and streptomycin resistance rates of *B. melitensis*were were 76.2%, 19.0%, 76.2%, 66.7%, and 4.8%, respectively. In contrast, 25.0%, 87.5%, 25.0%, and 37.5% of *B. abortus* were resistant to ciprofloxacin, erythromycin, imipenem, and rifampicin, respectively. All phenotypically resistant isolates have mutations in the rpoB gene linked to rifampicin resistance. Additionally, four isolates of *B. melitensis* that exhibited phenotypic resistance were found to have mutations in the gyrA and gyrB genes linked to ciprofloxacin resistance. In Egypt’s *Brucella* isolates, presence of these mutations reveals the molecular mechanisms underlying antibiotic resistance (Khan et al., 2019).

Another study by Dadar et al. used next-generation sequencing (NGS) technology and traditional phenotyping to assess AMR and virulence-associated variables in *Brucella* isolates obtained from people and animals in various parts of Iran. Their research identified *B. melitensis* as the most prevalent species among camels, small ruminants, and cows. There was only one human instance from which *B. abortus* was isolated.

For ampicillin-sulbactam, trimethoprim-sulfamethoxazole, colistin, and rifampicin, probable intermediate or resistant phenotypic patterns were discovered. All isolates had *mprF, bepG, bepF, bepC, bepE*, and *bepD* as identified by whole-genome sequencing (WGS; however, other conventional AMR genes were not found. The genomes of all *Brucella* isolates contained 43 genes linked to five virulence factors, and there was no variation in the distribution of these genes. One gene encoded the Rab2-interacting conserved protein A (*ricA*), 12 genes were linked to a type IV secretion system (*virB1-B12*), two were linked to proteins that contain the toll-interleukin-1 receptor (TIR) domain (*btpA, btpB*), and 27 genes were linked to lipopolysaccharide (LPS). One gene was linked to the synthesis of cyclic β-1,2 glucans (*cgs*). This is the first study to disclose virulence factors and molecular-based AMR in *Brucella* isolated from humans and several animal hosts in Iran. Notably, most of the antibiotics used to treat human brucellosis still have the ability to *in vitro* affect Iranian isolates of *B. abortus* and *B. melitensis*. There was no variation in the distribution of virulence-associated genes across all isolates, and WGS was unable to identify traditional AMR genes. However, it remains unclear why the genomes of resistant strains lack traditional AMR genes. Further research is needed to investigate the proteomic and transcriptome mechanisms underlying phenotypic resistance (Dadar et al., 2023).

In India, Ayoub et al. explored the genetic basis of AMR in *B. melitensis* strains. Twenty-four isolates from humans and animals were subjected to antimicrobial susceptibility testing and whole-genome sequencing. The results showed resistance to ciprofloxacin (16.67%), doxycycline (20.80%), rifampicin (16.67%), and cotrimoxazole (4.17%). All isolates had efflux-related genes, including *mprF, bepG, bepF, bepC, bepE*, and *bepD*, according to genome analysis; however, no traditional AMR genes were found. Interestingly, both resistant and susceptible isolates have mutations in key AMR-associated genes like *rpoB, gyrA*, and *folP*, indicating a complex genotype–phenotype link in AMR among *Brucella* spp. Furthermore, it was observed that both resistant and some susceptible isolates had mutations in efflux genes, suggesting that these genes may play a part in resistance mechanisms. Nevertheless, phenotypic resistance did not always correspond with changes in AMR-associated genes, indicating a complex basis for resistance (Ayoub et al., 2024).

Moreover, In Ulanqab, Inner Mongolia, China, a total of 85 *B. melitesis* isolates were collected by Liu et al. from humans, assessing their resistance to nine antibiotics. The examined isolates were all sensitive to ciprofloxacin, gentamicin, levofloxacin, minocycline, sparfloxacin, doxycycline, and tetracycline. Of the isolates, 1.0% (1/85) and 7.0% (6/85) showed resistance to rifampin and cotrimoxazole, respectively. However, single isolates displaying rifampin resistance did not show alterations in the *rpoB* gene (Liu et al., 2018).

In Bosnia and Herzegovina, Arapović prospectively analyzed the rates of resistance among human *Brucella melitensis* strains. 108 *B. melitensis* isolates from 209 patients with diagnoses from five medical centers were included in this study. The *B. melitensis* isolates’ resistance patterns to the 13 most widely used antibiotics were examined. Blood cultures from 111 (53.1%) of the 209 patients tested positive for *B. melitensis*. 84.3% of the 108 isolates under investigation exhibited resistance to trimethoprim-sulfamethoxazole. They found that *B. melitensis* was highly resistant to azithromycin. To comprehend the emergence of antibiotic resistance in human isolates of *B. melitensis*, further whole-genome sequencing research is required (Arapović et al., 2022).

A study performed in Norway provided a whole-genome sequencing and antimicrobial resistance in *Brucella melitensis* from a Norwegian perspective. The study analyzed the gene and protein sequences for seven known antibiotic resistance-associated genes (*rpoB, folA*, *folP*, g*yrA*, *gyrB*, *parC*, *parE*) and compared the sequenced isolates to the reference strain *B. melitensis* 16 M. Three different SNP variants were detected in *rpoB*, a gene associated with rifampicin resistance. The mutations detected in the *rpoB* gene in our data were located at nucleic acid position 1174 [392-Glu (GAG)◊Asp (GAC)], 1185 [629-Ala (GCG)◊Val (GTG)] and 2953 [985-Ala [GCC)◊Val (GTC)]. These alterations are different from mutations previously described as a cause of rifampicin resistance in *Brucella*. Additionally, the SNP alterations were not restricted to the four isolates phenotypically intermediate resistant to rifampicin based on gradient strip testing. The SNP changes therefore does not seem associated with rifampicin resistance. The observed SNP variants were however observed to be specific for certain lineages and sub-clusters based on WGS analysis; the SNP in position 1174 was detected in two related isolates within the Af clade, SNP in position 1185 was common for all isolates in the EM clade, and the SNP in position 2953 was restricted to the isolates in sub-cluster A in the EM clade. One mutation was detected in *folA*, a structural gene coding for dihydrofolate reductase and described to be involved in resistance mechanisms for trimethoprim. The detected SNP at position 73 [217-Arg (CGG)◊Leu (CTG)] was present in all isolates belonging to the Af clade in the current dataset. In *folP*, the gene coding for dihydrofolate synthase and associated with sulfamethoxazole resistance33, one SNP difference was detected compared to the reference strain at position 631 [211-Phe (TTC)◊Leu (CTC)].

Changes in the genes *gyrA*, *gyrB*, *parC* and *parE* were also detected. These genes are all known as fluoroquinolone-resistance determining genes, coding for DNA gyrase and DNA topoisomerase respectively. The described mutation in *gyrA* did not correspond with mutations related to fluoroqinolone resistance described earlier. The SNP detected in g*yrA* in position 1759 [599-Leu (CTG)◊Val (GTG)], was detected in all isolates in the EM clade, but was also detected in one isolate in the Af clade. The mutation in the *gyrB* gene was detected only in the reference strain *B. abortus* B19, and not in any *B. melitensis* isolates. Four SNP differences were detected in *parC*, of which two was only present in *B. abortus* (#21), and one SNP difference was detected in *parE*. The SNP difference in *parE* in position 27 [79-Asn (AAC)◊Ser (AGC)] was present in all isolates in the Af clade, and the SNP at position 799 [267-Arg (CGC)◊His (CAC)] in *parC* was present in a sub-cluster in the Af clade (Johansen et al., 2018).

## 7 Diagnostic tools of brucellosis

The therapeutic management and control of infection depend heavily on prompt and accurate diagnosis. Bacterial culture techniques and other serological approaches are the primary means of detection. These methods also aid in monitoring programs, herd screening, and the planning, control, and eradication tactics in diverse global locations.

### 7.1 Bacterial isolation

*Brucella* species can be diagnosed using a variety of techniques, although the most reliable ones are isolation and culture of the organism (Al Dahouk et al., 2003). The use of a selective medium, such as Farrell’s medium, is recommended because all *Brucella* strains develop somewhat slowly, and the specimens from which isolations are typically conducted or attempted are frequently highly contaminated (Pappas et al., 2006). A negative diagnosis may not be made until after a week of incubation, which typically lasts 72 hours. Fetal stomach fluid, spleen, liver, placenta, lochia, milk (particularly colostrum or milk within a week of calving), semen, and lymph nodes (supramammary for chronic and latent infections, and retropharyngeal for early infections) are among the specimens that can be used for *Brucella* isolation. *Brucella* colonies are high, translucent, convex, with complete boundaries, smooth, and radiant surfaces (Bedore and Mustefa, 2019). Under transmitted light, the colonies seem honey-colored. The ideal pH range for culture is between 6.6 and 7.4, while the ideal temperature range is between 20°C and 40°C. CO_2_ is necessary for the growth of certain *Brucella* species. A culture can only be deemed negative if no colonies form after two to three weeks of incubation, while typical colonies emerge after two to thirty days (Ashenafi et al., 2007).

### 7.2 Serological tests

Since the majority of brucellosis control and eradication programs rely on serological testing, these tests are essential for laboratory diagnosis. They are classified into two main categories: screening tests and confirmatory tests. Although a number of serological tests have been utilized for laboratory testing of brucellosis, no single test is practical for all epidemiological studies because of issues with sensitivity and/or specificity (Mert et al., 2003). The Rose Bengal plate test, indirect enzyme-linked immunosorbent assay, and serum agglutination test are the most often utilized serological assays for brucellosis (Luelseged, 2018). Due to its ease of use and apparent readability, the Rose Bengal plate test (RBPT) is the most popular screening method for brucellosis in both humans and animals. Personal experience, however, may influence how the RBPT results are interpreted (Cho et al., 2010).

RBT’s shortcomings include limited specificity in endemic areas, low sensitivity, especially in chronic patients, and prozones that cause strongly positive sera to look negative (Díaz et al., 2011). Another effective screening test for dairy cattle is the Milk RingTest (MRT) (Mohamand et al., 2014). Although MRT is a straightforward and efficient serological technique, it is limited to usage with cow’s milk (Mohamand et al., 2014). In a glass or plastic tube, a drop of hematoxylin-stained antigen is combined with a tiny amount of milk. MRT is highly ambiguous at the individual animal level, but it is applicable to the entire herd and provides a basic picture of the infection status. Its shortcomings include its incapacity to male animals and its decreased dependability in large herds (Luelseged, 2018).

One popular confirmatory test for brucellosis is the complement fixation test (CFT). The Organization for Animal Health (WOAH) recommends CFT as the reference test for international animal transit due to its high accuracy. It is used as a confirmatory test for *B. abortus, B. melitensis*, and *B. ovis* infections (Sam et al., 2012). CFT is typically utilized on sera that test positive for RBPT, but like RBPT, it is also heavily impacted by the overuse of the strain 19 vaccine, especially when sexually mature cows and heifers have received recent or repeated immunizations. Strict cutoff readings that indicate infection are nearly impossible to prescribe, especially when S19 vaccine reactions are involved due to their potential for abuse. Occasionally negative results in the early stages of infections, high cost and technical complexity are some of CFT’s drawbacks. Additional limitations include the test’s incapacity to be used with hemolyzed serum samples and the subjectivity of interpreting the sporadic direct activation of complement by serum (anti-complementary activity). Animals infected with species antigenically similar to *Brucella* can also provide false-positive results. As a common test for brucellosis diagnosis, the enzyme-linked immunosorbent assay (ELISA) has gained popularity (Al Dahouk and Nöckler, 2011). It is a valuable method for detecting *Brucella* antibodies in large populations and distinguishing between the acute and chronic stages of the illness. All four antibody classes (IgM, IgG1, IgG2, and IgA) can be identified with the ELISA method (Franco et al., 2007). The ELISA is an excellent control test in regions free of brucellosis and for survey testing in areas where vaccination has not been administered. However, it is difficult to use and cannot be used everywhere, particularly in areas where vaccination has been administered and is still not widely standardized (Al Dahouk and Nöckler, 2011).

One of the common serological assays for brucellosis diagnosis is the serum agglutination test (SAT) (Memish and Balkhy, 2004). It is straightforward to execute and does not require costly tools or extensive training. The total amount of IgM and IgG agglutinating antibodies is measured by SAT. The foundation of this test is the responsiveness of antibodies to *Brucella*’s smooth lipopolysaccharide. Applying a serial dilution of 1:2 through 1:64 to the blood samples can increase test specificity by removing excess antibodies that cause false-negative results due to the prozone effect (Al Dahouk and Nöckler, 2011). The incapacity to identify *B. canis* infections and the emergence of IgM immunoglobulin cross-reactions with *Salmonella urbana*, *Escherichia coli* O116 and O157, *Francisella tularensis*, and *Yersinia enterocolitica* O:9 are disadvantages of the serum agglutination test. By adding EDTA, 2-mercaptoethanol, or antihuman globulin, for example, some of these drawbacks can be addressed (Shemesh and Yagupsky, 2011).

### 7.3 Molecular tests

An *in vitro* method for amplifying nucleic acids, the polymerase chain reaction (PCR) is frequently employed in the diagnosis of infectious disorders. PCR is one of the most widely used tests for brucellosis diagnosis in humans nowadays (Queipo-Ortuño et al., 2008). The most widely used molecular methods for diagnosing brucellosis are PCR and/or its variations, which are based on the amplification of particular genomic sequences of the genus, species, or even biotypes of *Brucella* species (Dauphin et al., 2009). Compared with traditional PCR, real-time PCR is faster and more sensitive. Because PCR products do not require post-amplification handling, there is a reduced risk of contamination in the lab and false-positive results. *Brucella* cells can now be tested using real-time PCR techniques, according to recent studies (Al-Nakkas et al., 2005).

### 7.4 Nanotechnology methods

Flower-like gold nanoparticles labeled and silver deposition rapid vertical flow technology for highly sensitive detection of *Brucella* antibodies. The rapid vertical flow technique (RVFT) was discovered to identify brucellosis antibodies. It can successfully avoid the false negative problem in lateral flow assay. It is easy to use, with a quick time of 2–3 minutes visible to the naked eye and no special equipment. LPS were utilized to detect brucellosis antibodies to improve the procedure’s sensitivity. The advantages of the lateral flow immunoassay were kept while a single multipurpose buffer was developed in whole blood and other biological samples to enhance the sensitivity of serum antibody detection (Elrashedy et al., 2022).

## 8 Treatment of brucellosis

The stage of the disease (acute, subacute, or chronic), clinical severity, presence and types of focal disease (arthritis, epididymo-orchitis, spondylitis, endocarditis, neurobrucellosis, deep abscess, aortitis, etc.), targeted population groups (pregnant patients, lactating women, children under 8 years old), underlying conditions (immunosuppression, hepatic or renal failure), and the *Brucella* species (Alavi and Alavi, 2013; Tosatto et al., 2020) should all be taken into consideration when designing an anti-*Brucella* treatment regimen. Moreover, consideration should be given to antibiotic delivery techniques, drug availability, possible contraindications, and cost (Ariza et al., 2007).

Doxycycline, rifampin, trimethoprim/sulfamethoxazole (TMP–SMX), aminoglycosides (gentamicin or streptomycin, infrequently amikacin), and quinolones (ciprofloxacin or ofloxacin) are used to treat human brucellosis (Ariza et al., 2007). Ceftriaxone is currently a safe medication to use during pregnancy (Bosilkovski et al., 2019) and shows promise as a combination therapy for some *Brucella*r-related complications, such as neurobrucellosis, endocarditis, etc (Fatani et al., 2019). If there are no contraindications, doxycycline is the most successful treatment for brucellosis and forms a fundamental part of any therapeutic combination. Its MIC90 activity range is 0.004 to 1 mg/L. It is widely accessible, reasonably priced, and exhibits strong intracellular and exceptional activity in the acidic phagolysosomal environment (Solera, 2010).

In addition, rifampin is a first-line medication that has good bactericidal activity (MIC90:rifampin, 0.02–2.5 mg/L) (Bosilkovski et al., 2021) and great phagocytic cell penetration. Its anti-*Brucella* activity increases by 2–8 times at low pH (Akova et al., 1999). There have been reports of decreased susceptibility and even significant resistance to rifampin in *Brucella* over the past few decades (Hashim et al., 2014; Torkaman Asadi et al., 2017). When treating brucellosis, aminoglycosides are regarded as a significant class of antimicrobials. Their intracellular penetration is poor (Lecaroz, 2006), but their *in vitro* activity is good (MIC90: streptomycin, 0.125–8, average 2.5 mg/L; gentamicin, 0.25–2 mg/L) (Hall, 1990). *In vitro*, TMP-SMX exhibits excellent activity, adequate tissue, and intracellular penetration (MIC90: TMP-SMX, 0.38–8, average 1–2 mg/L) (Hall, 1990).

Though it can be used in alternate and three-drug regimens, TMP-SMX should not be the first line of treatment, except for pregnant women and children under the age of eight (Ariza et al., 2007). In certain parts of the world, *Brucella* have a high level of resistance to TMP-SMX (Irajian et al., 2016; Gu et al., 2020). Quinolones are alternative medications for the treatment of brucellosis because they have a low MIC90 (ofloxacin: 0.02–2.5 mg/L; ciprofloxacin: 0.06–2.0 mg/L), excellent intracellular penetration, and high tissue concentration (Falagas and Bliziotis, 2006). However, they have poor bactericidal activity in an acidic intracellular environment. Due to their high cost, lack of clinical experience, and the successful results seen with previous quinolones, newer fluoroquinolones, such as levofloxacin, are not advised for routine use in brucellosis (Ariza et al., 2007).

The doxycycline–streptomycin regimen, when compared with the doxycycline–gentamicin regimen, manifested equal efficacy and overall similar tolerability (Roushan et al., 2010). Fluoroquinolones, TMP-SMX, and their combinations with rifampin or doxycycline for six weeks are examples of alternative therapies (Skalsky et al., 2008). A combination regimen of rifampin and quinolones has shown encouraging outcomes in several studies for the treatment of brucellosis (Hashemi et al., 2012). When both were given for six weeks, the combination of doxycycline, rifampin, and levofloxacin produced a higher incidence of side effects and a much lower relapse rate than the doxycycline–rifampin regimen (Hasanain et al., 2016).

## 9 Brucellosis-related economic losses

Economic losses associated with brucellosis in livestock have been observed in different nations (Santos et al., 2013; Singh et al., 2015). All data indicate that brucellosis causes significant global economic losses in livestock health, production, and public health (cost of treatment and productivity loss in humans), even though estimates of the costs associated with brucellosis infections are still restricted to particular countries. For example, epidemiological surveys in India revealed that livestock brucellosis caused a median estimated economic loss of US $3.4 billion (Mantur and Amarnath, 2008; Singh et al., 2018).

According to another official report, bovine brucellosis causes about $600 million in annual economic damages in Latin America. It has been estimated that farms impacted by brucellosis see a 20–30% reduction in milk production (Herrera et al., 2008; Grace et al., 2020). However, only a few nations accurately report the losses they suffer from brucellosis. For example, Argentina loses up to US $60 million a year (Samartino, 2002), India loses a median of US $3.4 billion for cattle, sheep, and goats (Singh et al., 2015), Egypt loses US $9.8 million, the United States loses US $30 million, Brazil loses about US $448 million, and Kyrgyzstan loses about US $10.6 million (Dadar et al., 2021).

In Nigeria, brucellosis in small ruminants resulted in annual economic losses of US $3.2 million two decades ago. However, the brucellosis eradication programs may be quite expensive in developing countries (Zhang et al., 2018). For instance, the national brucellosis eradication program in the United States was estimated to have cost $3.5 billion between 1934 and 1997. Numerous economic ramifications for the livestock sector and public health prompted attempts to manage brucellosis in low-income and endemic nations using various strategies that will be covered in the following sections (Mcdermott et al., 2013).

## 10 Control and prevention measures against *Brucella*

According to Perez-Sancho et al. (2015), the World Health Organization (WHO) has identified brucellosis as one of the seven neglected zoonotic diseases that contribute significantly to poverty in poor nations. Furthermore, a brucellosis outbreak control program is beneficial for dairy herd maintenance. According to Olsen and Stoffregen (2005), brucellosis control programs can employ all or any of the following strategies: vaccination, testing and removal methods, and/or sanitation. Furthermore, the most cost-effective method of controlling brucellosis is to vaccinate cattle between the ages of 4 and 12 months and animals older than 12 months (Dadar et al., 2021). However, according to Olsen and Stoffregen (2005) (Olsen and Stoffregen, 2005), immunization alone is insufficient to eradicate brucellosis in any host species. The live vaccination strains of *B. abortus* currently used more frequently to prevent brucellosis in cattle include RB51 and S19 (Hou et al., 2019). Furthermore, the *Brucellamelitensis* REV-1 vaccination is the most efficient method for eliminating and controlling brucellosis in small ruminant animals, both young and adult. This method is the most effective when it comes to extended or nomadic husbandry and situations where small ruminants have a high frequency of brucellosis (Godfroid et al., 2013). The success of *B. melitensis* control programs seems to depend on vaccine efficacy and coverage, which are essential for preventing *Brucella* infections in small ruminants (Beauvais et al., 2016). Numerous elements must be assessed by the planned control program, including knowledge of local and regional differences in brucellosis epidemiological patterns in animals, cross-sectoral brucellosis epidemiological coordination and surveillance, husbandry practices, infrastructure support, community awareness, and social customs. A test-and-slaughter approach can be used to manage bovine brucellosis on dairy farms in nations with a low prevalence of the illness (Tesfaye et al., 2011). According to reports, other preventive measures including vaccinating female bovines and certifying herds free of brucellosis, are also successful methods for controlling the disease (Blasco and Molina-Flores, 2011; Avila-Granados et al., 2019). Strict national surveillance programs are therefore required to identify affected herds and enable the implementation of any ensuing preventative and remedial actions (Rivera et al., 2002). Ultimately, a combination of strategies is required for the effective control of brucellosis in animals, including animal surveillance using serological testing to identify infected animals, preventing the spread of brucellosis to herds of animals that are not infected, and removing animal carriers of the bacteria, such as dogs, cats, and mice, from the herd to eliminate the sources of infection (Kiros et al., 2016). For long-term eradication and control programs to be implemented, farmers’ cooperation and support are essential. Therefore, through ongoing education and training initiatives, veterinary organizations should raise farmers’ awareness of prevention tactics and transmission pathways. Other crucial prerequisites include the availability of resources for prevention and suitable veterinary care (Dadar et al., 2021).

## 11 Metal acquisition

Every form of life depends on metals. In biological processes such as DNA replication, transcription, respiration, precursor biosynthesis, and reactions to oxidative stress, metal cofactors have both structural and catalytic roles. The first-row transition metals—cobalt, nickel, copper, manganese, and iron—are necessary for most living things. Because of their homeostasis systems, organisms only accumulate the metals necessary to meet their physiological needs. These systems include efflux systems, chaperones, transfer, and storage proteins that keep these metals in non-toxic or unreactive forms, as well as transcriptional and translational regulators that strictly control the expression of the genes encoding these metal import, export, and storage (Summers, 2009; Palmer and Skaar, 2016).

Metal homeostasis mechanisms in mammals guard against metal toxicity and prevent invading microorganisms from establishing a productive infection. Iron sequestration, for example, is a well-documented technique used by mammals to inhibit microbial pathogen reproduction (Nairz et al., 2010). In the extracellular environment, iron-binding proteins such as transferrin and lactoferrin bind firmly to iron that has not been integrated into host proteins. Most iron is present as Fe^3+^ at physiological pH in this oxidizing environment. The amount of ‘free’ Fe^3+^ in blood and tissue fluids is believed to be <10–^18^ M. During infection, hepcidin inhibits ferroportin from releasing iron from nutritional sources and senescent or damaged erythrocytes into the bloodstream. This limits the availability of iron in the host’s extracellular environment. This so-called ‘hypoferremic response’ is considered a crucial component of innate immunity (Nemeth et al., 2004; Weiss, 2005).

Iron is present in the host’s intracellular environment as a dynamic equilibrium between Fe^2+^ and Fe^3+^. The ratio depends on the redox status, pH, and activity of cellular ferric reductases and ferroxidases (Anderson and Vulpe, 2009). Three mechanisms have been identified for mammals to deprive microbial pathogens such as *Brucella*, which dwell within phagosomal compartments in host macrophages, of iron. All three of these mechanisms are considered critical components of the host’s immunological response to infection. The first involves the natural resistance-associated macrophage protein (Nramp1) (Cellier et al., 2007). This protein enters macrophages’ phagosomal membranes and removes divalent cations like Fe^2+^ and Mn^2+^. Macrophages triggered by interferon γ (IFNγ) have lower numbers of transferrin receptors on their surface, reducing iron flow through the host cells. Finally, while iron release from host cells via ferroportin is often inhibited during the hypoferremic response, the activity of ferroportin in infected macrophages increases, resulting in active iron efflux from these cells (Nairz et al., 2007).

*Brucella* strains require iron, manganese, zinc, and magnesium transporters for wild-type virulence.

*Brucella* strains require iron, manganese, and magnesium for optimal growth *in vitro*. Phenotypic evaluations of defined mutants indicate that efficient zinc transport is also necessary for virulence in experimentally infected animals (Roop, 2012).

### 11.1 Iron transport

Iron is a co-factor for many different proteins because of its chemical flexibility. Iron is presumably necessary for the activity of a wide variety of *Brucella* proteins. Catalase, aldolase (Roop, 2012), and CobG, an enzyme involved in the production of cobalamin (vitamin B12), are a few examples of which this requirement has been experimentally confirmed (Schroeder et al., 2009).

#### 11.1.1 The mammalian host is an Fe-deprived environment

Because *Brucella* strains live mostly near their mammalian hosts, they face a unique barrier in obtaining enough iron to meet their physiological requirements (Roop et al., 2009). This is due in part to the fact that the great majority of iron in mammals is not readily available because it is integrated into proteins as heme, Fe-S clusters, or mononuclear or dinuclear Fe centers (Corbin et al., 2008). Another factor to take into account is that the soluble and physiologically active form of iron, Fe^2+^, can combine with reactive oxygen species to produce harmful hydroxyl radicals (OH−). Consequently, mammals’ Fe homeostasis systems keep the amounts of “free” iron in their tissues at levels also referred to as siderocalin. This prevents the action of siderophores, which are small molecular weight chelators secreted into the environment by microbes to capture and transport iron (Yang et al., 2002; Sia et al., 2013). Furthermore, the liver generates the peptide hormone hepcidin (Hp), which causes ferroportin to degrade and stops iron from being released into the bloodstream from the liver and spleen (Michels et al., 2015). Thus, the already low quantities of free Fe accessible in the extracellular environment of mammals are further reduced by the combined actions of lactoferrin, calprotectin, and HP.

AAMs absorb large amounts of proteins, including iron and heme, due to their function in scavenging injured cells and tissue components (Cairo et al., 2011). Though greater ferroportin (Fp) activity in AAMs keeps intracellular Fe levels from becoming hazardous, AAMs have bigger intracellular labile Fe pools than CAMs. Additionally, less of this Fe is deposited in ferritin in AAMs than in CAMs, despite increased Fe flow through AAMs. It is yet unknown if the larger labile Fe pool in AAMs plays a role in *Brucella* strains choosing to inhabit these phagocytes during persistent infections. Regarding the possible sources of iron that *Brucella* strains in their mammalian hosts may access, three additional points are worth taking into account: (a) the recycling of iron from mammalian erythrocytes; (b) the increased uptake of heme-containing proteins by AAMs; and (c) the intracellular trafficking of *Brucella* strains in host cells.

#### 11.1.2 Fe acquisition by *Brucella*

##### 11.1.2.1 Siderophore production

**Figure 6:**
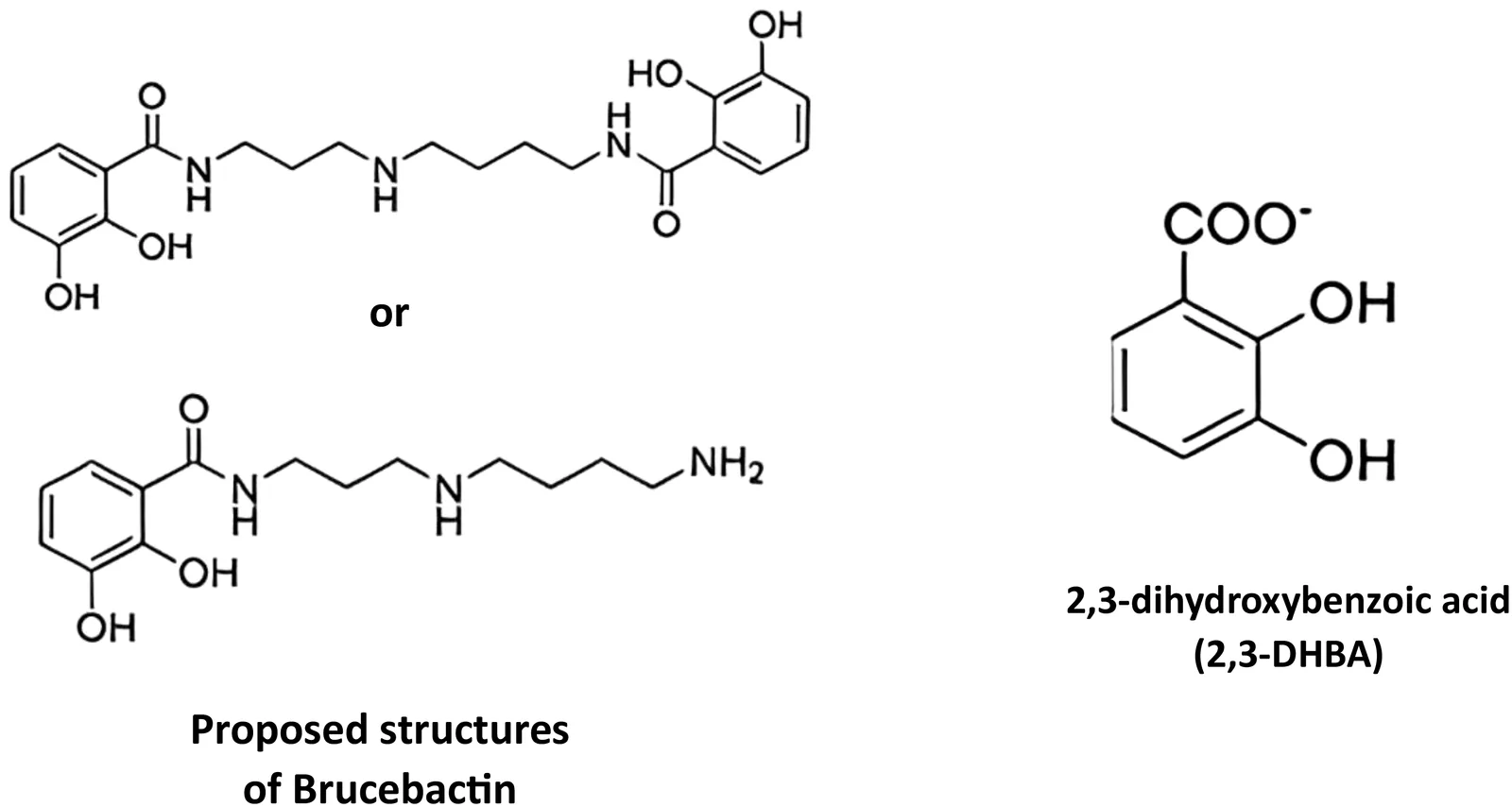
Siderophores produced by *Brucella*.

Microbes release siderophores, which are low molecular weight chelators, into the surrounding environment in order to absorb iron (Raymond and Dertz, 2014). When exposed to iron deficiency, strains of *Brucella* create two catechol siderophores: brucebactin, which is a compound based on 2,3-DHBA, and 2,3 dihydroxybenzoic acid (2,3-DHBA) (González Carreró et al., 2002). The exact structure of brucebactin is currently unclear due to its instability in the lab. However, according to the molecular characteristics of the enzymes encoded by the genes responsible for these siderophores, brucebactin is most likely a monocatechol made up of 2,3-DHBA connected to either an amino acid or a polyamine (Bellaire et al., 2003b). Although siderophore synthesis is not necessary for the persistence of *Brucella* strains in host macrophages, experimental data indicates that it is crucial to the pathogenicity of these bacteria in the gravid ruminant reproductive tract. For example, pregnant goats (Bellaire et al., 1999) and cattle (Bellaire et al., 2003b) do not experience abortion when exposed to a *B. abortus* dhbC mutant, which does not produce 2,3-DHBA or brucebactin. However, in the mouse model of chronic infection, this mutant, along with isogenic *B. abortus* mutants that generate 2,3-DHBA but are unable to convert it to brucebactin, exhibits wild-type pathogenicity (González Carreró et al., 2002; Michelle et al., 2002). The structures of *Brucella* siderophores are shown in **Figure 6**.

The ability of *B. abortus* to use erythritol as its preferred carbon and energy source has been proposed as one reason for the apparent differential demand for siderophore formation in the bovine reproductive system. This four-carbon sugar alcohol is produced in large quantities by ruminant placental trophoblasts in the later stages of pregnancy. It has been suggested that the *Brucella*’s ability to effectively use this carbon source is connected to their virulence in pregnant ruminants. *B. abortus* 2308 exhibits a significantly higher requirement for iron while growing in the presence of erythritol than when growing with other easily utilizable carbon and energy sources, according to *in vitro* investigations (Bellaire et al., 2003a; Jain et al., 2011). It has been suggested that the formation of siderophores is crucial in providing this strain with the iron required to support the broad and quick bacterial reproduction in placental trophoblasts that results in abortion (Bellaire et al., 2003a).

When exposed to iron shortage *in vitro*, not all strains of *B. abortus* and *B. melitensis* produce catechol siderophores (Roop, 2012). Therefore, it will be crucial to clarify the relationship between siderophore synthesis and erythritol metabolism in *Brucella* strains and conduct conclusive research to ascertain whether or not this relationship accounts for the severe attenuation that the *B. abortus* dhbC mutant exhibits in pregnant ruminants. Determining whether or whether siderophore synthesis is necessary for the virulence of different *Brucella* strains in a range of natural hosts, both pregnant and non-pregnant, will also be crucial.

##### 11.1.2.2 Siderophore transport systems in *Brucella*

**Figure 7:**
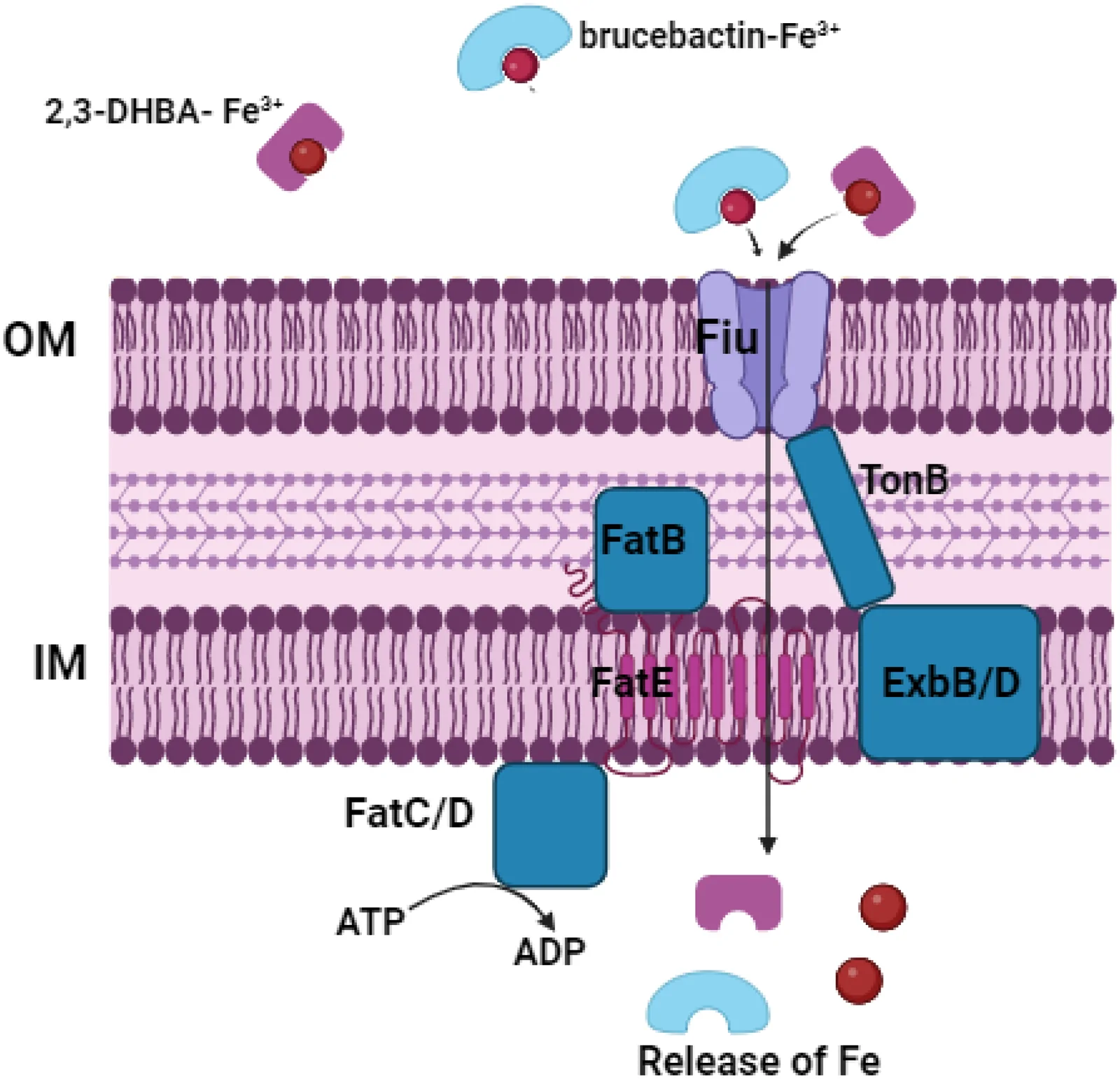
Siderophores-Iron transporters in *Brucella*. OM, outer membrane; IM, inner membrane.

Due to their size, Fe^3+^-siderophore complexes require energy to transport into the cytoplasm of bacteria. In Gram-negative bacteria, transport across the outer membrane is typically facilitated by ‘gated’ porins, which obtain the energy they need to drive this transport from the ExbBD-TonB system (Noinaj et al., 2010). These Fe^3+^-siderophore complexes are then bound by specific periplasmic binding proteins which direct them to cytoplasmic ABC-type permeases that mediate their passage across the cytoplasmic membrane (**Figure 7**). Once in the cytoplasm, Fe^3+^ is released from the siderophore by its reduction to Fe^2+^ and/or degradation of the siderophore (Harrington and Crumbliss, 2009). Two genetic loci involved in Fe^3+^-siderophore transport have been identified in *Brucella*—fatBDCE and exbBD-tonB. Published studies have shown that *B. abortus* fatB and *B. melitensis*fatC and exbB mutants cannot use brucebactin and 2,3-DHBA, respectively, as Fe sources *in vitro* (González Carreró et al., 2002; Danese et al., 2004). In contrast, the identity of the genes that encode the TonB-dependent OM protein that transports Fe^3+^-brucebactin across the outer membrane is presently unclear.

#### 11.1.3 Heme as an Fe source for *Brucella* strains

It was demonstrated that exogenous heme could rescue the heme auxotrophy of a *B. abortus* hemH mutant, and the parental 2308 strain can transport the intact heme molecule (Roop Ii et al., 2017b). This was an important finding because as noted previously, the intracellular trafficking pattern of the *Brucella* in host macrophages places these bacteria in an environment where heme is conceivably a relevant Fe source.

**Figure 8:**
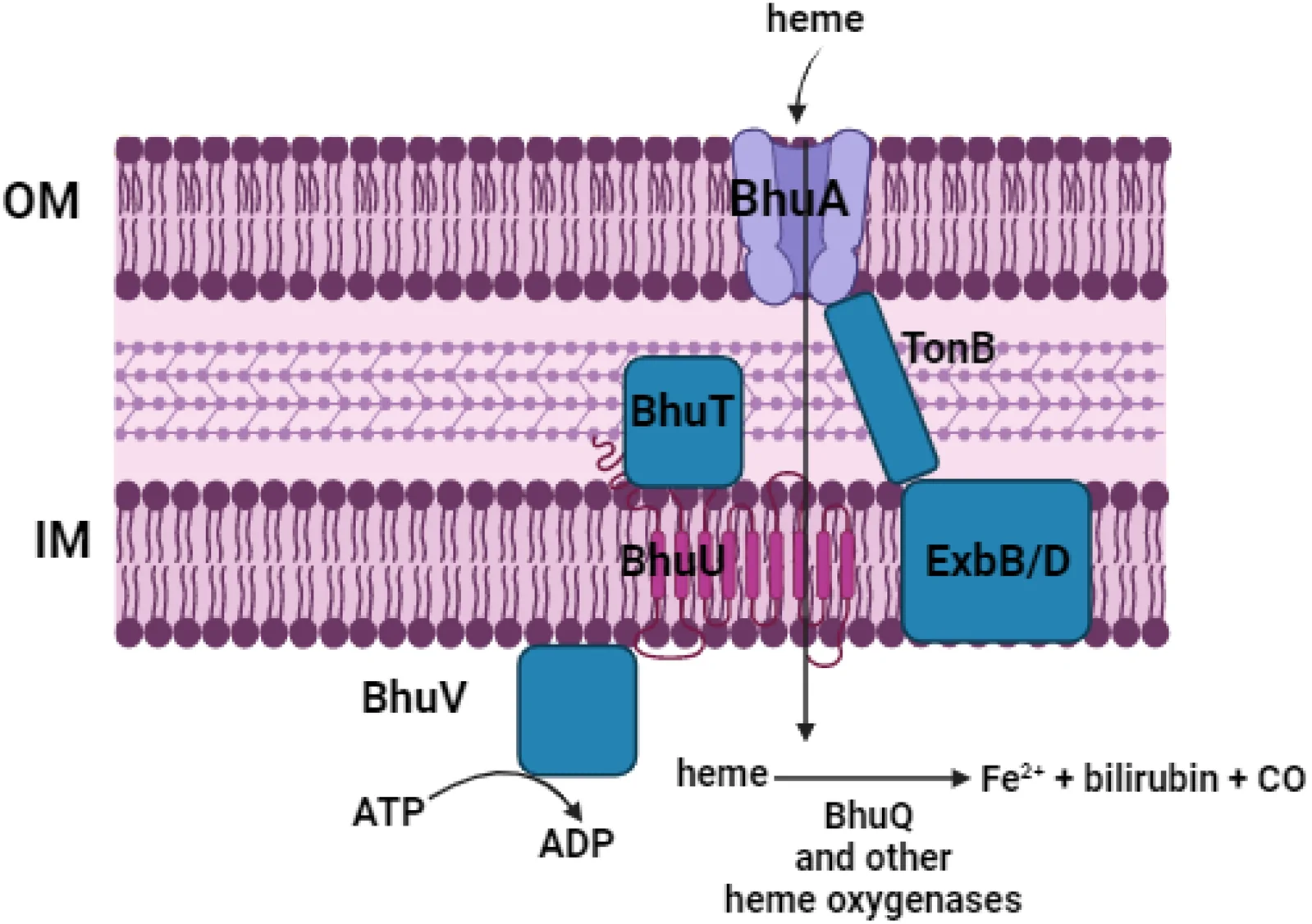
Heme transporter in *Brucella*. OM, outer membrane; IM, inner membrane.

One of the main roles of mammalian macrophages is the destruction of damaged and senescent erythrocytes and the recycling of the iron expelled from these cells (Bratosin et al., 1998). To provide the growing fetus with iron, ruminant placental trophoblasts also consume and break down maternal erythrocytes (Anderson et al., 1986). During both procedures, a significant amount of heme is delivered into these host phagocytes. In *in vitro* tests, *B. melitensis*16M and *B. abortus* 2308 may both use heme as an iron source (Bellaire et al., 2003a; Danese et al., 2004; Paulley et al., 2007). The periplasmic binding protein-dependent ABC-type transporter, which is made up of the proteins BhuT, U, and V, and the TonB-dependent outer membrane protein, BhuA, mediate heme transport (**Figure 8**). *Brucella* strains seem to have well-preserved genes that encode these proteins (Roop, 2012). Heme oxygenase is also present in *Brucella* strains (Puri and O’Brian, 2006). This enzyme, which we have designated BhuO (Roop, 2012), presumably degrades heme after it has been delivered into the cytoplasm, enabling the *Brucella* to exploit it as an iron source. In experimentally infected mice, an isogenic bhuA mutant derived from *B. abortus* 2308 exhibits notable attenuation (Paulley et al., 2007), indicating that the ability to transport heme is a crucial virulence factor.

It is yet to be experimentally established if heme consumption contributes significantly to the virulence of other *Brucella* strains or in wild hosts. The heme not integrated into cellular proteins in human cells is actively transported to the endoplasmic reticulum (ER), where heme oxygenase might destroy it due to its possible toxicity (Taketani, 2005). Numerous interactions between phagosomes and the host cell ER result in the membrane-bound vacuoles, often called replicative *Brucella*-containing vacuoles or rBCVs, in which the *Brucella* proliferate in host macrophages (Celli et al., 2003). After interacting with the ER exit sites, the rBCVs eventually fuse with the ER (Celli et al., 2005). Microscopically and in placental trophoblasts from experimentally infected ruminants, extensive contacts between rBCVs and the host cell ER have also been detected (Anderson et al., 1986). Therefore, it will also be crucial to ascertain how the interactions of the rBCVs with the host cell ER affects the availability of heme as an iron source for *Brucella* strains during their intracellular residence in macrophages and placental trophoblasts in order to better understand the host-pathogen interactions in brucellosis.

#### 11.1.4 Siderophore-independent transport of Fe^3+^ in *Brucella*

**Figure 9:**
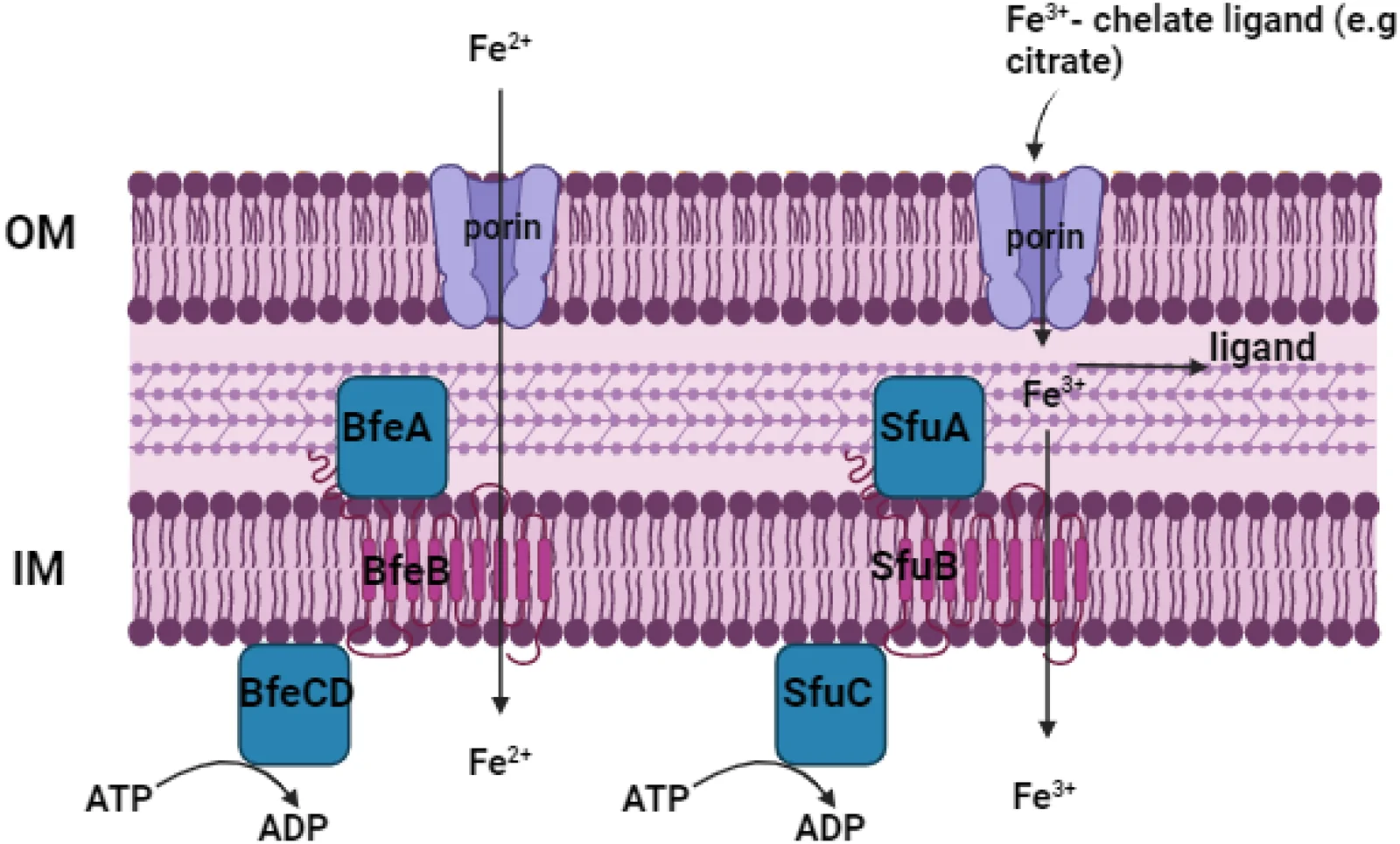
Iron transporter in *Brucella*. OM, outer membrane; IM, inner membrane.

Fe^3+^-siderophore complexes generally require energy for transport across the outer membrane of Gram-negative bacteria (Noinaj et al., 2010), but Fe^3+^ bound to low molecular weight chelators such as citrate can diffuse across the outer membrane via porins, where specialized Fe^3+^-specific periplasmic protein-dependent ABC transporters can capture this Fe^3+^ and transport it across the cytoplasmic membrane. Such transporters include the Sfu, Afu, and Yfu (Roop Ii et al., 2017b) systems described in *Serratia, Actinobacillus*, and *Yersinia*, respectively (**Figure 9**). Two sets of genes predicted to encode Sfu-type Fe^3+^ transporters have been described in *Brucella* (Jenner et al., 2009), but the corresponding gene products’ roles in Fe transport and/or virulence are unknown.

#### 11.1.5 Detoxification of excess intracellular Fe by *Brucella* strains

Bacteria must not only be able to import enough Fe to meet their physiological needs but also have a means of maintaining cellular levels of unincorporated Fe below ‘toxic’ levels. One ‘indirect’ mechanism that they use is to tightly regulate the genes encoding their Fe import systems so that they only import Fe when cellular levels fall below a certain threshold. How *Brucella* strains employ this strategy to prevent toxicity will be discussed in detail in a subsequent section. The other two more ‘direct’ mechanisms that bacteria employ to prevent Fe toxicity are to (a) convert excess intracellular Fe^2+^ into a ‘non-toxic’ form (e.g., Fe^3+^) for storage in proteins such as bacterioferritin and Dps; and (b) to export excess intracellular Fe^2+^ from the cell.

#### 11.1.6 Bacterioferritin and Dps

Bacterioferritins (Bfrs) are large, 24 subunit proteins that with 12 heme groups form hollow spheres in bacterial cells (Andrews, 2010). These proteins have distinctive ferroxidase centers that convert soluble Fe^2+^ to insoluble Fe^3+^, which is then stored as 2Fe(O)OH in the interior of these spherical proteins. Each Bfr can store up to 4500 atoms of Fe^3+^, and this Fe^3+^ can be converted to Fe^2+^ and released into the bacterial cytoplasm as needed to replenish depleted Fe levels. By converting the highly reactive Fe^2+^ to the less reactive Fe^3+^ and sequestering it away from the other cytoplasm components, Bfr serves as a depot for excess Fe and prevents this excess Fe from reaching toxic levels. Dps is another spherical protein found in bacteria that oxidizes Fe^2+^ to Fe^3+^ and stores the insoluble Fe^3+^ in its interior (Andrews, 2010). Although Dps, like Bfr, is considered a member of the ferritin-like superfamily of proteins, it has three important structural and functional differences when compared to Bfr. First, Dps is made up of 12 subunits, and is thus smaller than Bfr, and consequently can store less Fe (e.g., 500 atoms) than Bfr. The ferroxidase site in Dps is also different from that of Bfr, and Dps uses H_2_O_2_ instead of O_2_ to catalyze the oxidation of Fe^2+^. In addition, many, but not all bacterial Dps proteins, bind non-specifically to DNA. Based on these properties and the phenotypic analysis of bacterial dps mutants, it has been proposed that the major physiologic function of Dps is cellular defense against oxidative stress rather than Fe storage (Chiancone and Ceci, 2010). *Brucella* strains produce both Bfr (Almirón and Ugalde, 2010) and Dps (Kim et al., 2014). Phenotypical analysis of a defined mutant indicates that Bfr plays a role in Fe metabolism in *B. abortus* 2308. For instance, a bfr mutant is more sensitive to Fe deprivation and has reduced levels of intracellular Fe than the parent strain (Almirón and Ugalde, 2010). The biological function of Dps, on the other hand, is presently unclear. Although the *dps* gene is strongly regulated by the general stress response sigma factor RpoE1 in *B. abortus* 2308 (Kim et al., 2014), a *B. abortus dps* mutant exhibits wild-type resistance to H_2_O_2_ *in vitro* assays. Considering the possibility that Dps could conceivably play a compensatory role to Bfr in terms of Fe storage, it will be important to evaluate the Fe storage capabilities and virulence properties of *Brucella* bfr *dps* double mutants to adequately evaluate their respective biological functions.

### 11.2 Manganese transport

One high-affinity Mn transporter, MntH, is produced by *Brucella* strains, and the Mn-responsive transcriptional regulator Mur controls the expression of the related gene (Anderson et al., 2009). Mn-dependent enzymes are essential to *Brucella* strains’ fundamental physiology and pathogenicity, according to phenotypic examination of a range of mutations. These bacteria may also modify their cellular Mn level as a defense against oxidative stress (Roop Ii et al., 2017c). A *B. abortus mntH* mutant is significantly attenuated in the mouse model of chronic infection. It is yet unknown what causes this attenuation. The *B. abortus mntH* mutant has lower Mn superoxide dismutase activity than the parental strain, but an isogenic *sodA* mutant only shows mild attenuation in mice (Martin et al., 2012), suggesting that the *mntH* mutant’s severe attenuation is not caused by lower SodA activity. Although the relationship between Mn^2+^ transport and *virB* expression has not been studied, the *B. abortus mntH* mutant also shows aberrant expression of the genes encoding the Type IV secretion machinery (Anderson et al., 2009). One possible explanation for this relationship is that orthologs of the (p)ppGpp synthetase/hydrolase known as Rsh (Dozot et al., 2006), Manganese-dependent enzymes are necessary for both the induction of the stringent response in *Brucella* and the production of VirB (Pappas et al., 2006).

According to recent genetic and biochemical studies, *E. coli* can replace Fe^2+^ in important metabolic enzymes like ribulose-5-phosphate epimerase (Rpe), a key enzyme in the pentose-phosphate pathway, by increasing the intracellular ratio of Mn^2+^:Fe^2+^. *Escherichia coli* also shows increased *mntH* expression in response to exposure to H_2_O_2_ (Anjem et al., 2009). Since Mn^2+^ does not take part in Fenton reaction like Fe^2+^ does, this substitution shields Rpe from damage caused by H_2_O_2_. *In vitro* exposure of *B. abortus* 2308 to H_2_O_2_ also increases the expression of *mntH*, and *in vitro* experiments show that an isogenic *mntH* mutant is more sensitive to exposure to H_2_O_2_ than the original 2308 strain (Anderson et al., 2009). In order to protect these proteins from H_2_O_2_-mediated damage, it will be crucial to ascertain whether *Brucella* strains possess the same ability as *E. coli* to replace Fe^2+^ in metabolic enzymes with Mn^2+^.

### 11.3 Zinc transport

Many bacteria, including *Brucella* species, depend on zinc as a micronutrient. In *Brucella* strains, zinc is necessary for the correct operation of multiple critical enzymes involved in the production of amino acids and oxidative stress tolerance (Andreini et al., 2006). Zinc is also a crucial cofactor for the type IV secretion system effector protein RicA (Rab2 interacting conserved protein A) and the virulence-associated transcriptional regulatory protein MucR. *Brucella* strains have a high affinity zinc uptake mechanism termed ZnuABC that preferentially imports zinc because of its significance in their biology. On the other hand, the *Brucella* also encode a zinc export mechanism, ZntA, which aids in the intracellular detoxification of excess zinc due to the hazardous potential of free zinc cations (Sheehan et al., 2015). Zinc uptake and export systems are tightly regulated by two zinc-responsive regulatory proteins, Zur and ZntR, which regulate transcription of the znuABC and zntA genes, respectively (Clapp et al., 2011). Crucially, effective pathogenesis in animal models of *Brucella* infection depends on the appropriate homeostasis of zinc levels in the virus. The systems that control zinc uptake and export in *Brucella* strains will be described in this chapter, along with their function in virulence, the genetic control of zinc homeostasis, and zinc’s involvement as a cofactor for key enzymes in *Brucella* strains (Caswell, 2017). SodC is necessary for the *Brucella’s* complete pathogenicity and the bacteria’s high resistance to exogenous O_2_. In the periplasmic region of many Gram-negative bacteria, including several dangerous organisms, SodC, a periplasmic superoxide dismutase (SOD), is in charge of detoxifying exogenously produced superoxide (i.e., O_2_^−^). The SodC protein is frequently referred to as Cu-Zn SOD because bacterial SodC proteins require copper and zinc cofactors for proper enzymatic function, while the zinc cation appears to play a crucial structural role (Caswell, 2017).

### 11.4 Nickel transport

It has been demonstrated that urease is one of the few bacterial enzymes that needs nickel as a cofactor (Li and Zamble, 2009). According to Bandara et al. (2007) and Sangari et al. (2007), this enzyme is necessary for the pathogenicity of *B. abortus* 2308 and *B. suis* 1330 in mice when these strains are introduced orally, but not when they are given peritoneally. In mammalian cell cultures, *B. suis* and *B. abortus* urease mutants display wild-type pathogenicity. Urease is not necessary for intracellular survival in eukaryotic cells, but it helps the *Brucella* withstand the extremely low pH they experience during passage through the stomach and gastrointestinal tract following ingestion, according to the theory put up to explain these observations. Although NikABCDE and NikKMLQO, two nickel transporters, have been found in *Brucella* (Jubier-Maurin et al., 2001b; Sangari et al., 2010), how these transporters contribute to virulence is unclear. An isogenic *nikA* mutant generated from *B. suis* 1330 exhibits wild-type pathogenicity in the human monocytic cell line THP-1, even though *nikA* expression is increased in this strain during intracellular replication in J774 cells (Jubier-Maurin et al., 2001a). It will be crucial to evaluate the virulence characteristics of *Brucella* strains deficient in either the NikABCDE or NikKMLQO transporter, or both, in cultured macrophages and in mice infected by both the intraperitoneal and oral routes to better understand the necessity of nickel transport by *Brucella* strains in the host.

### 11.5 Magnesium transport

Bacterial cells contain large quantities of magnesium (mM). It is a structural and enzymatic co-factor for many cellular proteins and is crucial for preserving the structural integrity of ribosomes and cell membranes (Moomaw and Maguire, 2008). For example, Mg^2+^ is necessary for the activity of erythritol kinase, the enzyme that catalyzes the initial stage of erythritol catabolism in *Brucella* strains.

**Figure 10:**
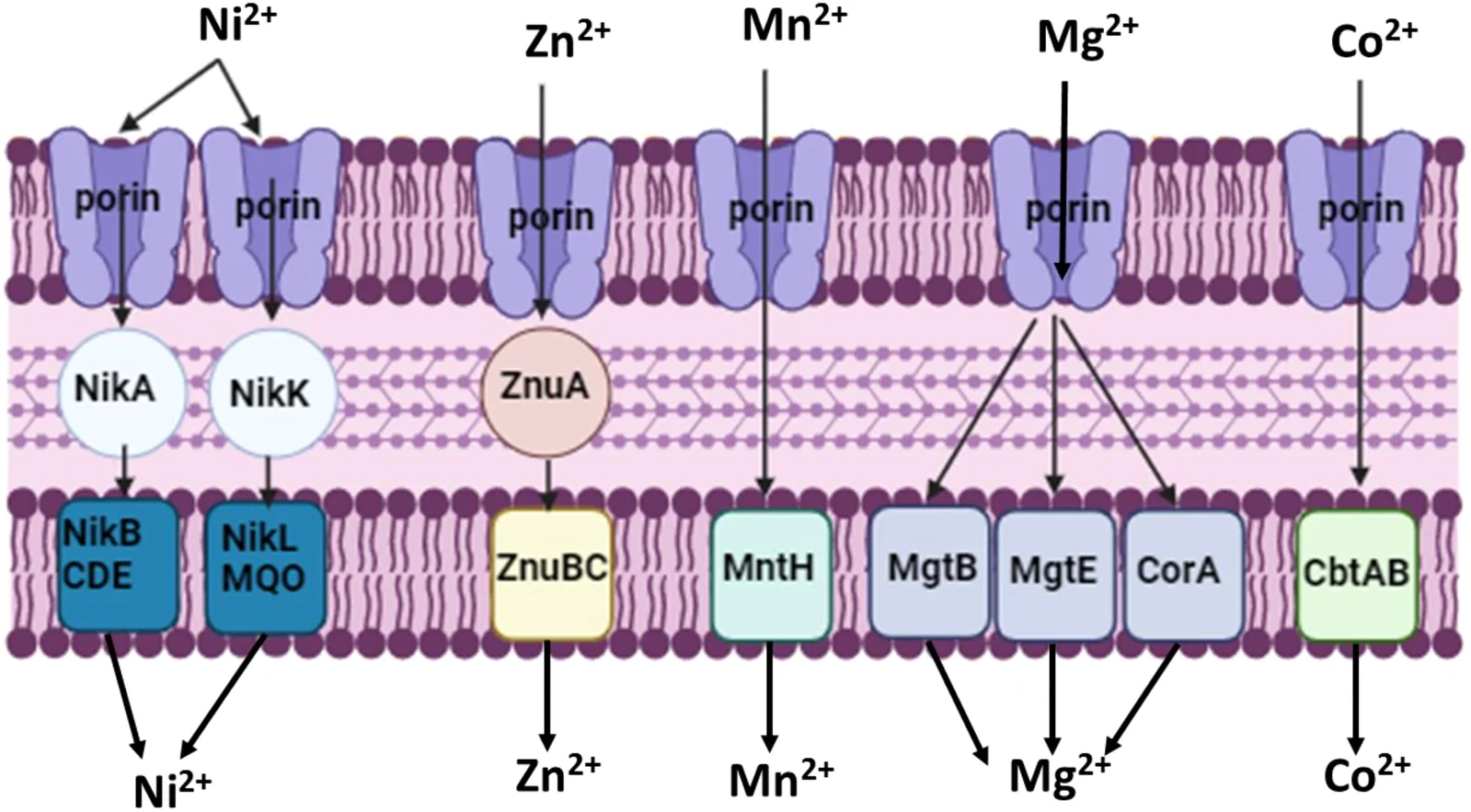
Nickel, zinc, manganese, magnesium and cobalt transporters in *Brucella*. OM, outer membrane; IM, inner membrane.

Virulence in *Brucella* strains has been genetically related to homologs of two genes involved in magnesium transport in other bacteria. Salmonella is the greatest example of the activity of the bacterial P-type ATPase MgtB as a magnesium transporter (Smith et al., 1993). During a screening of signature-tagged transposon mutants obtained from *B. melitensis*16M for attenuation in experimentally infected mice, a *B. melitensis mgtB* mutant was discovered (Lestrate et al., 2000). Remarkably, when cultivated in magnesium-limited media, this mutant showed no signs of growth defects. This implies that the *Brucella* have several magnesium transport mechanisms, just like other bacteria and as shown in **Figure 10**. A *B. suis mgtC* mutant does not grow well in a magnesium-restricted medium and exhibits significant attenuation in the murine macrophage-like J774 cell line (Lavigne et al., 2005), although the exact function of MgtC in magnesium transport has not been determined (Günzel et al., 2006; Alix and Blanc-Potard, 2007). Adding MgCl2 to the cell growth media can help mitigate this attenuation to some extent.

### 11.6 Cobalt transport

Many bacteria, including *Brucella*, require cobalt as a micronutrient because it is a necessary component of cobalamin (vitamin B12) (Warren et al., 2002). Additionally, cobalamin is necessary for the activity of several key bacterial enzymes, including the ribonucleotide reductase NrdJ (Taga and Walker, 2010) and the methionine synthase MetH (Roop Ii et al., 2017a). In mice and cultured mammalian cells, *B. suis* and *B. melitensis* mutants with defects in the cobalamin biosynthesis pathway are attenuated (Delrue et al., 2004). *Brucella* strains have an intact cobalamin biosynthesis pathway (Lundqvist et al., 2009). These infection models also attenuate a *B. melitensis metH* mutant (Lestrate et al., 2000). Between the cobalamin biosynthesis genes *cobQ* and *cobU* in *Brucella* are two genes that are anticipated to encode a CbtAB-type Cotransporter. The idea that the *Brucella* cbtA and B genes express a high-affinity Co transporter is supported by their location and the fact that they are located downstream of a cobalamin-responsive riboswitch (Rodionov et al., 2003), but this function has not been experimentally verified (Roop Ii et al., 2017a).

### 11.7 Metalloregulators and metal storage/detoxification proteins p

The prevention of toxicity resulting from the excessive buildup of these crucial micronutrients depends on proteins that directly contribute to metal homeostasis. Three transcriptional regulators—Irr (Martínez et al., 2005; Martínez et al., 2006), DhbR (Anderson et al., 2008), and Mur (Menscher et al., 2012)—have been identified as controlling the expression of *Brucella* metal acquisition genes. DhbR is an AraC-type transcriptional regulator that activates the transcription of the siderophore biosynthesis genes in *B. abortus* 2308 in response to Fe^3+^-siderophore levels in the external environment; Mur controls the expression of the gene encoding the Mn^2+^ transporter MntH in response to cellular Mn^2+^ levels; and Irr is an iron-responsive transcriptional regulator that governs the genes involved in iron acquisition and iron metabolism. Bacterioferritin (Bfr), a protein that accumulates and detoxifies intracellular iron, is also produced by certain strains of *Brucella* (Denoel et al., 1995; Almirón and Ugalde, 2010). Only Irr and Bfr have had their involvement in virulence investigated thus far. Though neither *B. abortus* nor *B. melitensis-bfr* mutants show attenuation in cultured human primary explant macrophages (Denoel et al., 1997), J774 or HeLa cells (Almirón and Ugalde, 2010), or experimentally infected mice (Denoel et al., 1997), a *B. abortus irr* mutant is attenuated in the mouse model (Anderson et al., 2011).

## 12 Vaccines

The most effective way to stop human infection is to control animal brucellosis. Research and scientific studies to develop brucellosis vaccines have been underway since the early 1900s. Inactivated, live-attenuated, and rough-attenuated vaccines are all part of the brucellosis vaccine development process. Inactivated vaccines were first developed as a disease prevention strategy. To prevent brucellosis, however, more immunologically effective live attenuated vaccines eventually took their place. The several kinds of brucellosis vaccinations and their effectiveness are listed below.

### 12.1 Live attenuated vaccines

In order to improve safety and immune responses, recent developments have concentrated on modified live attenuated vaccines with removed virulence genes. For instance, a mutant of the *B. melitensis* TcfSR promoter (Li et al., 2015) and the *B. melitensis* 16M hfq mutant strain (Zhang et al., 2013) showed considerable protection and minimal interference with serodiagnostic assays. Although they are sensitive to polymyxin B (Wang et al., 2014), other possible vaccines, including the M5-90ΔwboA mutant and 6MΔ*wzt*, have shown decreased pathogenicity and enhanced defense mechanisms. Similar to the *B. ovisabcBA* (*BoabcBA*) vaccine (Costa et al., 2020), the *2308DNodVDNodW* rough vaccine from the virulent *B. abortus* 2308 strain provides a strong immune response against the *B. melitensis* strain 16M. *Brucella* double gene knock-out vaccine strain MB6 Δbp26ΔwboA (RM6) was constructed and evaluated by Shi et al. the researchers have found that the RM6 strain had good proliferative ability and stable biological characteristics *in vivo* and *in vitro*. Moreover, it had a favorable safety profile and elicited specific immune responses in mice and sheep (Shi et al., 2022).

### 12.2 Subunit vaccines

Subunit vaccines exhibit promise in terms of non-infectious, non-viable, and safety. Nevertheless, they are not very good at simulating the spread of genuine illnesses (Ficht et al., 2009). Subunit vaccines have the advantage of being safe, but in order to produce strong immunity and protect cattle from brucellosis, they need to be administered in numerous booster doses and utilizing a variety of antigens, adjuvants, and delivery systems. However, due to associated expenditures, this strategy could not be commercially viable (Perkins et al., 2010). Regretfully, no viable subunit vaccination against brucellosis has been created in spite of countless attempts (Zimmermann and Curtis, 2019).

### 12.3 Vaccines based on nanoparticles

Oral vaccines containing nanoparticles and the *Brucella* vaccine produced antibody responses, including IgM, mucosal IgA, and IgG, in animal model studies. In animal investigations, these vaccinations have shown significant benefits, including a stronger Th1–Th17 immune response (Abkar et al., 2017). However, human brucellosis cannot be prevented by nanoparticle-based vaccinations because of the possible danger of disease transmission (Maleki et al., 2019). These vaccines’ primary disadvantages include their toxicity, limits in terms of antigen loading and manufacturing, and their less-than-ideal capacity to activate the immune system (Al-Halifa et al., 2019).

### 12.4 DNA vaccines

In the fight against brucellosis, DNA-based *Brucella* vaccines have proven to be both safe and effective. These vaccines’ capacity to produce antigens and integrate CpG patterns results in strong cellular immune responses. Simple storage conditions are another benefit of DNA-based vaccinations. They have important gene sequences that are essential to *Brucella* species’ intracellular survival (Hu et al., 2009). However, as compared to live-attenuated vaccines, DNA-based vaccines do not offer as much protection. Studies show no discernible changes in the expression of IL-4, IL-10, or IFN-γ, suggesting an immunological response to DNA-based vaccinations (Rosinha et al., 2002).

### 12.5 Vector vaccines

Using *Brucella* as a delivery vehicle, live vector-based vaccines have become a successful way to deliver a variety of antigens, both homologous and heterologous. By multiplying inside host cells and creating several copies of the *Brucella* antigen, these genetically engineered vaccines are designed to elicit an antigen-specific T-cell immune response (Al-Mariri et al., 2002).

### 12.6 Recombinant peptides

One promising strategy for preventing and controlling brucellosis is the use of recombinant peptides as vaccinations. Conventional vaccinations, such as the Rev-1 vaccine, have drawbacks, such as the potential to cause abortion in fetuses and disrupt diagnostic procedures. Recombinant peptides, on the other hand, provide safer and more precise substitutes (Cassataro et al., 2005).

## 13 Conclusion

In conclusion, a disease’s successful management, including individualized treatments and early detection, depends critically on understanding its biological components. Although the development of vaccines is informed by continuous research on disease processes, the length of time needed emphasizes the need of ongoing medication discovery. Additionally, more research is required using natural hosts and inoculation routes that resemble those seen in nature. In order to better understand the fundamental characteristics of *Brucella* pathogenicity, mice have proven invaluable. *Brucella* strains in their natural hosts may require different virulence determinants, and these determinants may be needed at different phases of the disease cycle in these hosts (e.g., chronic infection versus abortion and fetal pathology in ruminants). Finally, most research assessing the role of *Brucella* metal acquisition in virulence has been conducted in the mouse model of chronic infection, which is used to gauge the strains’ capacity to endure and proliferate in host macrophages. However, the mouse model results may not necessarily anticipate how a mutant would behave in the wild host, particularly in pregnant ruminants, as the experiments using *B. abortus* siderophore biosynthesis mutants clearly show. Whether or not these bacteria are living in macrophages, or placental trophoblasts may affect the sources of iron (such as Fe^2+^, Fe^3+^, and heme or heme-containing proteins) and other metals that are available as well as the metabolic needs of the intracellular *Brucella* for these metals. Future research must, therefore, evaluate the contribution of metal acquisition genes to virulence in a range of natural and experimental hosts, both pregnant and non-pregnant.
